# Inferring epidemiological parameters from phylogenies using regression-ABC: A comparative study

**DOI:** 10.1371/journal.pcbi.1005416

**Published:** 2017-03-06

**Authors:** Emma Saulnier, Olivier Gascuel, Samuel Alizon

**Affiliations:** 1 Laboratoire Maladies Infectieuses et Vecteurs: Ecologie, Génétique, Evolution et Contrôle - UMR CNRS 5290, IRD 224 et UM, Montpellier, France; 2 Institut de Biologie Computationnelle (IBC) and Laboratoire d'Informatique, de Robotique et de Microélectronique de Montpellier (LIRMM) - UMR 5506, CNRS et UM, Montpellier, France; 3 Unité de Bioinformatique Evolutive, C3BI - USR 3756, Institut Pasteur et CNRS, Paris, France; Imperial College London, UNITED KINGDOM

## Abstract

Inferring epidemiological parameters such as the *R*_0_ from time-scaled phylogenies is a timely challenge. Most current approaches rely on likelihood functions, which raise specific issues that range from computing these functions to finding their maxima numerically. Here, we present a new regression-based Approximate Bayesian Computation (ABC) approach, which we base on a large variety of summary statistics intended to capture the information contained in the phylogeny and its corresponding lineage-through-time plot. The regression step involves the Least Absolute Shrinkage and Selection Operator (LASSO) method, which is a robust machine learning technique. It allows us to readily deal with the large number of summary statistics, while avoiding resorting to Markov Chain Monte Carlo (MCMC) techniques. To compare our approach to existing ones, we simulated target trees under a variety of epidemiological models and settings, and inferred parameters of interest using the same priors. We found that, for large phylogenies, the accuracy of our regression-ABC is comparable to that of likelihood-based approaches involving birth-death processes implemented in BEAST2. Our approach even outperformed these when inferring the host population size with a Susceptible-Infected-Removed epidemiological model. It also clearly outperformed a recent kernel-ABC approach when assuming a Susceptible-Infected epidemiological model with two host types. Lastly, by re-analyzing data from the early stages of the recent Ebola epidemic in Sierra Leone, we showed that regression-ABC provides more realistic estimates for the duration parameters (latency and infectiousness) than the likelihood-based method. Overall, ABC based on a large variety of summary statistics and a regression method able to perform variable selection and avoid overfitting is a promising approach to analyze large phylogenies.

## Author summary

Given the rapid evolution of many pathogens, analysing their genomes by means of phylogenies can inform us about how they spread. This is the focus of the field known as “phylodynamics”. Most existing methods inferring epidemiological parameters from virus phylogenies are limited by the difficulty of handling complex likelihood functions, which commonly incorporate latent variables. Here, we use an alternative method known as regression-based Approximate Bayesian Computation (ABC), which circumvents this problem by using simulations and dataset comparisons. Since phylogenies are difficult to compare to one another, we introduce many summary statistics to describe them and take advantage of current machine learning techniques able to perform variable selection. We show that the accuracy we reach is comparable to that of existing methods. This accuracy increases with phylogeny size and can even be higher than that of existing methods for some parameters. Overall, regression-based ABC opens new perspectives to infer epidemiological parameters from large phylogenies.

## Introduction

To control epidemics, we must understand their dynamics. Classical analyses typically rely on prevalence or incidence data [1, 2], which correspond to the total number of reported cases, and the number of newly reported cases through time, respectively. By combining such data with epidemiological models, one can estimate key parameters, such as the basic reproduction number (*R*_0_), which is the number of secondary cases generated by an infectious individual in a fully susceptible host population. A robust and rapid estimation of epidemiological parameters is essential to establish appropriate public health measures [1, 3]. As a result, inference methods in epidemiology are under rapid development [4–7].

With the advent of affordable sequencing techniques, infected individuals can now be sampled in order to sequence genes (or even the complete genome) of the pathogen causing their infection. In the case of outbreaks, this sampling can represent a significant proportion of infected hosts [8, 9]. A time-scaled phylogeny can readily be inferred from virus sequences with known sampling dates. Such a “genealogy” of infections bears many similarities with a transmission chain and potentially contains information about the spread of the epidemic. This idea was popularised by Grenfell et al. [10], who coined the term “phylodynamics” to describe the hypothesis that the way rapidly evolving parasites spread leaves marks in their genomes and in the resulting phylogeny.

Obtaining quantitative estimates from phylogenies of sampled epidemics remains a major challenge in the field [11, 12]. In most studies, epidemiological parameters are inferred using a Bayesian framework based on a likelihood function that describes the probability of observing a phylogeny given a demographic model for a set of parameter values. This model is sometimes referred to as the “tree prior” [13]. Epidemiological dynamics were first captured in the tree prior by using coalescent theory and assuming an exponential growth rate of the epidemic [14], or more flexible variations in the effective population size over time (i.e. effective prevalence) [15–17].

More recently, progress has been made in deriving tree priors relevant to epidemiological models (see [18] for a review). In 2009, Volz et al. [19] managed to express the likelihood function of SIS (for “Susceptible-Infected-Susceptible”) and SIR (for “Susceptible-Infected-Removed”) epidemiological models using coalescent theory, thus allowing for the estimation of *R*_0_. One year later, Stadler [20] derived the likelihood function of a phylogeny using the birth-death process with incomplete sampling. The method was then extended to other epidemiological models and allows for the inference of both *R*_0_ and the duration of the infection [21, 22].

It is now possible to compute the likelihood of a tree under most SIR type models using the coalescent approach [23, 24]. Other developments have combined state-of-the-art techniques in epidemiological modelling, for instance particle filtering, with the coalescent model for phylodynamics inference [23–25]. The success of these tree priors was made possible by advances in computing power, and the generalisation of computationally intensive techniques to explore the parameter space, such as Markov Chain Monte Carlo (MCMC) procedures [26]. Many of the tree priors and procedures described above, are implemented in the software packages BEAST [13] and BEAST2 [27].

Very recently, the Phylogenetics And Networks for Generalized HIV Epidemics in Africa (PANGEA-HIV) consortium reported on the ability of several phylodynamics methods to infer the parameters of a detailed individual-based model of HIV transmission in Sub-Saharan Africa, using only sampled sequences or phylogenies [28]. Of the five methods they compared, four were likelihood-based. The accuracy achieved by some of the methods, especially that involving the structured coalescent, was impressive, with some correlations between estimates and true values that were greater than 90%. However, this accuracy came at cost in terms of computing power (“roughly 1 week of computation time on a 64-core machine of 2.5Ghz processors per analysis” for the structured coalescent on the PANGEA-HIV data [28]), because they rely on MCMC techniques.

One of the five PANGEA-HIV methods was based on Approximate Bayesian Computation (ABC). ABC is a likelihood-free method that proposes to bypass the difficulty in computing (and even sometimes formulating) the likelihood function, by performing simulations and comparing the simulated and “target” data, usually via distances computed on summary statistics [29–32].

The basic ABC algorithm, called rejection [33], consists in retaining a small fraction of simulations that are close to the target in view of the computed distance. These constitute the final posterior distribution of the parameters. Over the last decade, several improvements of the rejection algorithm have been proposed. ABC-MCMC consists in searching in the prior parameter space more efficiently by using MCMC-like approaches [34]. Sequential Monte Carlo (ABC-SMC) methods adjust the posterior distribution obtained by rejection by re-sampling parameters from the posterior and thus iterating the rejection process until convergence [35, 36]. Regression-ABC uses the simulations selected by rejection to learn a regression model (linear or not), which is then used to adjust the posterior distribution initially obtained by rejection [33, 37]. Importantly, regression-ABC has the advantage of being potentially less computationally intensive and also less sensitive to the curse of dimensionality of the set of summary statistics than the ABC-MCMC or ABC-SMC methods [37].

In epidemiology, ABC has been shown to infer parameters from genetic data as accurately as and more efficiently than a likelihood-based method implemented in BEAST [38]. This study did not involve phylogenies and, to our knowledge, ABC has only been applied to phylodynamics in two studies [39, 40]. As shown in the first of these studies, this lack of enthusiasm for ABC could be due to the fact that the approach can be sensitive with respect to the choice of summary statistics and requires careful calibration of the tolerance parameter [39]. More recently, an ABC-MCMC algorithm using a tree shape kernel distance was developed [40]. This was the only likelihood-free method in PANGEA-HIV, but it produced the results with the widest confidence intervals [28].

In this article, we introduce a new ABC phylodynamics approach with two essential features. First, since phylogenies are complex objects, we use a large number of summary statistics to describe them, whereas existing ABC phylodynamics studies either use only a few of these [39] or a functional distance [40]. Second, we use regression-ABC with built-in variable selection, whereas existing methods in phylodynamics rely on MCMC-like techniques [39, 40].

The article is structured as follows. We first present the methodology (epidemiological models, tree simulation methods, computed summary statistics, and the data sets and inference methods used for the comparisons). We then analyze the location of the epidemiological information in the phylogeny. Lastly, we show that the accuracy of the estimates obtained using our regression-ABC with the LASSO approach is comparable to that based on the likelihood function. Our regression-ABC even outperforms these methods when estimating the host population size in the SIR model from large phylogenies. The accuracy of regression-ABC also increases with phylogeny size, suggesting that this method becomes more valuable for larger datasets.

## Materials and methods

### Compartmental models

**Fig 1:**
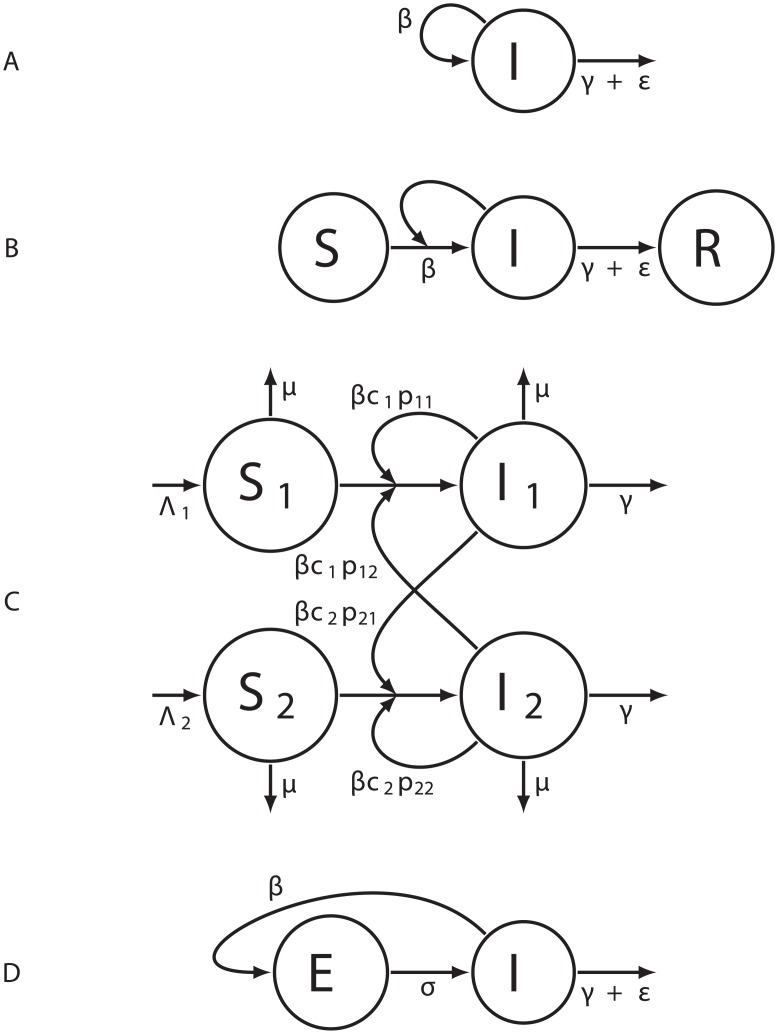
The epidemiological models. (A) The Birth-Death (BD) model. (B) The Susceptible Infected Removed (SIR) model. (C) The Susceptible Infected with Differential Risk (SI-DR) model. (D) The Birth-Death model including an Exposed class (BDEI). The four compartments correspond to susceptible (*S*), exposed (*E*), infectious (*I*) and removed (*R*) individuals. In BD and BDEI models, new infections arise at a constant (“birth”) rate *β* per infectious individual. In SIR and SI-DR models, the number of new infections depends on the number of susceptible individuals, the transmission rate *β* and the number of infectious individuals. In the SI-DR model, it also depends on the contact rates *c*_*i*_ associated with each risk group *i* = {1; 2}, and an assortativity term *p*_*ij*_ (*j* = {1; 2}). In both SIR and SI-DR models, the total host population size is assumed to be constant (*N*). In all models, infections end (i.e. “die”) at a rate *γ*. All models, except the SI-DR model, account for sampling that occurs at a rate *ε*. The SI-DR model accounts for demography (new susceptible individuals arise at a rate Λ_*i*_ and all individuals die at a rate *μ*).

We considered four epidemiological models: a Birth-Death (BD) model (Fig 1a), a Susceptible-Infected-Removed (SIR) model without demography (i.e. with a constant host population size, Fig 1b), a Susceptible-Infected with Differential-Risk (SI-DR) model and a Birth-Death model with an Exposed class (BDEI, Fig 1c).

These compartmental models are defined by ordinary differential equation (ODE) systems (see [40] for the SI-DR model and S1 Text for the three other models).

In these models, individuals susceptible to the pathogen become infected after contact with infectious individuals and successful transmission, which occurs at an overall transmission rate *β* [2], except for the SI-DR model [40] where the transmission rate is equal to *β* *c*_*i*_ *h*_*ij*_ depending on the risk groups of the “infectee” (*i* = {1; 2}) and the “infector” (*j* = {1; 2}). In the latter model, *c*_*i*_ is the contact rate of the individuals belonging to risk group *i* and the *h*_*ij*_ are the elements of an assortativity matrix (which [40] refers to as an “homophily” matrix) that describes the propensity of individuals from risk group *i* to have contact with individuals from risk group *j* (see [40] for more details on the computation of this matrix).

Following infection, individuals either become infectious immediately (BD, SIR and SI-DR models) or at a rate *σ* after a latency period in the Exposed class (BDEI model). They are then “removed” (i.e. recover with a lifelong immunity or die) at a rate *γ*. Lastly, they can be sampled, at a rate *ε*. By sampling, we mean that the pathogen is sequenced from the patient. Because sampling generally leads to treatment or at least to behavioral changes, we assumed that infected individuals are also “removed” after sampling. This assumption is commonly made in phylodynamics [21, 41, 42] and we kept it here to facilitate comparisons. However, it could easily be relaxed. The sampling proportion *p* is defined as the ratio of the sampling rate (*ε*) over the total removal rate (*γ* + *ε*).

The critical difference between BD models and the SIR model, lies in the transmission rate per infected individual λ(*t*): this rate is constant in BD models (λ(*t*) = *β*), but it depends on the susceptible population size in the SIR model ($\lambda \left(t\right)=\beta \frac{S(t)}{N}$, where *S*(*t*) is the number of susceptible individuals at time *t* and *N* is the effective population size). In other words, the SIR model assumes that the effective host population has a fixed size *N* and is initially fully susceptible (*S*(*t* = 0) = *N*). The susceptible population is depleted as the epidemic spreads (*S*(*t* > 0) < *N*) and this depletion decreases the speed of the spread of the epidemic (λ(*t* > 0) < λ(*t* = 0)).

In the SI-DR model used in [40], the number of new infections also depends on the susceptible population size, but there is no sampling because the model assumes that the sampling dates are known. The SI-DR model also accounts for demography since all individuals die at a rate *μ* and susceptible newborns of risk group *i* appear at a rate Λ_*i*_.

Our overall goal was to infer a vector of epidemiological parameters *θ*, from time-scaled phylogenies. For reasons related to method comparison, the composition of *θ* depends on the model:

- $\theta_{\text{BD}}=\left\{R_{0}=\frac{\beta }{\gamma +\varepsilon };\;d_{I}=\frac{1}{\gamma +\varepsilon }\right\}$,
- $\theta_{\text{SIR}}=\left\{R_{0}=\frac{\beta }{\gamma +\varepsilon };\;d_{I}=\frac{1}{\gamma +\varepsilon };\;N=S+I+R\right\}$,
- *θ*_SI-DR_ = {*c*_1_; *β*; *γ*; *N* = *S* + *I*}; *c*_2_, *μ*, *ρ* and *f* being fixed,
- $\theta_{\text{BDEI}}=\left\{R_{0}=\frac{\beta }{\gamma +\varepsilon };\;d_{E}=\frac{1}{\sigma };\;d_{I}\right\}$.

Contrary to the likelihood-based phylodynamics methods [8, 41, 42], we did not attempt to infer the sampling proportion using ABC, since only two out of the three parameters (*β*, *γ* and *ε*) are identifiable in the epidemiological models that account for sampling (see S1 Text) [43].

### Simulation of sampled transmission trees

The compartmental models described above are deterministic continuous-time models. However, whatever method is used (likelihood-based or not), epidemiological parameter inference requires taking the stochasticity of events at the individual level into account.

A time-scaled phylogeny of an epidemic can be viewed as a sampled transmission tree in which each branching represents a transmission and each leaf represents a sampled infected individual. There are several ways to simulate sampled transmission trees from epidemiological models. They all involve two processes: the simulation of the trajectory of the epidemic (i.e. the chronology of epidemiological events) and the construction of the sampled transmission tree based on this trajectory. In this study, we used two tree simulation approaches that can be applied to a wide variety of epidemiological models.

The first approach is implemented in the software MASTER [44] and is based on Gillespie’s direct method [2, 45] also known as the Stochastic Simulation Algorithm (SSA). This algorithm enables epidemiological models to be converted into event-driven models. A great advantage of the SSA is that there is an exact correspondence between the stochastic simulations and the deterministic ODE-based model. With this approach, trees are generally simulated alongside the trajectory, that is, through a forward-in-time birth-death process, where each birth in the tree corresponds to a transmission and each death corresponds to an end of infection with or without sampling. Unless the epidemiological model includes sampling as an event, MASTER produces full transmission trees. The computational complexity of this method is linear with respect to the total event count (C) with an additional time penalty associated with the tree update [44]. For the BD and the SIR models, C is the sum of the numbers of birth and death events. To obtain a sampled transmission tree of *n* leaves simulated assuming a sampling proportion *p* with either model, we need to simulate a full transmission tree composed of $\frac{n}{p}$ leaves (and $\frac{n}{p}\;-\;1$ internal nodes). Thus we need $C=(2\;\frac{n}{p}\;-\;1)$ events (births and deaths) to be performed. Gillespie’s SSA complexity is then in O(C), where C is at most proportional to $\frac{n}{p}$ with large n, for both models.

The second approach has been implemented in the rcolgem R package [23, 24]. In this approach, epidemiological models are translated into continuous-time stochastic models to simulate trajectories. Trees are simulated afterwards based on trajectories, through a backward-in-time coalescent-like process. The coalescent approach assumes that sampling dates are known, which means the epidemiological models do not require assumptions about the sampling process. With careful implementation and reasonable approximation, the trajectory can be generated in a time that is proportional to the simulated epidemic duration (*t*_*end*_ − *t*_0_) over the chosen time step (*δt*), and the tree can be built in a time that is proportional to its size (*n*). This approach becomes valuable when $C>(\frac{t_{end}-t_{0}}{\delta t}\;+\;n)$, *n* representing the number of leaves. This can be the case, for instance, when simulating large trees with very sparse sampling or for epidemiological models more complex than the SIR model, where the number of events does not depend only on the tree size and sampling proportion.

We used the MASTER-like approach for the BD, SIR and BDEI models, which all include sampling, and the rcolgem R package for the SI-DR model. Note that we implemented our own SSA instead of using MASTER to facilitate the addition of constraints on the simulations (see below).

### Summary statistics

Sampled transmission trees are complex objects. Therefore, we used summary statistics to compare them and capture the epidemiological information they may contain. We decided to compute many summary statistics to capture as much information as possible. This was motivated by the fact that there is no consensus in the field regarding which summary statistics to use. Importantly, our decision was made possible by the existence of efficient regression models that perform variable selection and can be combined to ABC (see below). Overall, we used 83 summary statistics, which we grouped into three “families” to better identify where the epidemiological information is in the phylogeny: branch lengths (Table 1), tree topology (Table 2) and Lineage-Through-Time (LTT) plot (Table 3) [46].

**Table 1:** Summary statistics based on branch lengths (bl set). ^•^ Statistics computed on three time-based parts of the tree. Internal branches belong respectively to the first (*k* = 1), second (*k* = 2) or third (*k* = 3) part of the tree if they end before the first, second or third delimitation, respectively. ^÷^ Ratios between each piecewise statistic related to internal BL and the same statistic computed on all external BL.

Notation	Description
*max_H*	Sum of the branch lengths between the root and its farthest leaf
*min_H*	Sum of the branch lengths between the root and its closest leaf
*a_BL_mean*	Mean length of all branches
*a_BL_median*	Median length of all branches
*a_BL_var*	Variance of the lengths of all branches
*e_BL_mean*	Mean length of external branches
*e_BL_median*	Median length of external branches
*e_BL_var*	Variance of the lengths of external branches
*i_BL_mean_[k]*^•^	Piecewise mean length of internal branches
*i_BL_median_[k]*^•^	Piecewise median length of internal branches
*i_BL_var_[k]*^•^	Piecewise variance of the lengths of internal branches
*ie_BL_mean_[k]*^÷^	Ratio of the piecewise mean length of internal branches over the mean length of external branches
*ie_BL_median_[k]*^÷^	Ratio of the piecewise median length of internal branches over the median length of external branches
*ie_BL_var_[k]*^÷^	Ratio of the piecewise variance of the lengths of internal branches over the variance of the lengths of external branches

**Table 2:** Summary statistics based on the tree topology (topo set).

Notation	Description
*colless*	Sum for each internal node of the absolute difference between the number of leaves on the left side and the number of leaves on the right side [47]
*sackin*	Sum for each leaf of the number of internal nodes between the leaf and the root [48]
*WD_ratio*	Ratio of the maximal width (*W*) over the maximal depth (*D*), where the depth of a node characterizes the number of branches that lies between it and the root, and the width *w*_*d*_ of a tree at a depth level *d* is the number of nodes that have the same depth *d* [49]
Δ*w*	Maximal difference in width $\Delta w={\text{max}}_{d=0}^{D-1}\left(\right|w_{d}-w_{d+1}\left|\right)$ [49]
*max_ladder*	Maximal number of internal nodes in a ladder which is a chain of connected internal nodes each linked to a single leaf, divided by the number of leaves [49]
*IL_nodes*	Proportion of internal nodes In Ladders [49]
*staircaseness_1*	Proportion of imbalanced internal nodes that have different numbers of leaves between the left and the right side [49]
*staircaseness_2*	Mean ratio of the minimal number of leaves on a side over the maximal number of leaves on a side, for each internal node [49, 50]

**Table 3:** Summary statistics based on the LTT plot (ltt set). ^•^ Computed on three part of the tree. Consecutive steps up respectively to the first (*k* = 1), second (*k* = 2) or third (*k* = 3) part of the tree if the second steps happens before the first, second or third delimitation, respectively.

Notation	Description
*max_L*	Maximal number of lineages
*t_max_L*	Time at which the maximal number of lineages is observed
*slope_1*	Linear slope between the origin and the maximal number of lineages
*slope_2*	Linear slope between the maximal number of lineages and the last leaf event
*slope_ratio*	Ratio of the *slope_1* over the *slope_2*
*mean_s_time*	Mean time between two consecutive down steps (mean sampling time)
*mean_b_time_[k]*^•^	Piecewise mean times between two consecutive up steps (piecewise mean branching times)

Since branching occurs throughout the phylogeny at a rate that varies over time (the number of infected and susceptible hosts vary in the SIR model), we designed all the summary statistics related to branching and internal branches (linking two internal nodes) in a piecewise manner (Table 1). We temporally cut the tree into three equal parts: internal branches belong respectively to the first, second or third part of the tree, if they end before the first ($\frac{1}{3}max\_H$), second ($\frac{2}{3}max\_H$) or third (*max*_*H*) delimitation, respectively, where *max*_*H* represents the height of the farthest leaf. We only computed global summary statistics (on the whole tree) to describe sampling events and external branches (linking internal nodes to the leaves).

It is known that the topology of a phylogeny can be driven by processes such as immune escape [10]. Moreover, it has been shown recently that different transmission patterns can result in quantitatively different phylogenetic tree topologies. In particular, heterogeneity in host contact can influence the tree balance [49]. That is why we also used phylogenetic topological indexes as summary statistics (Table 2).

**Fig 2:**
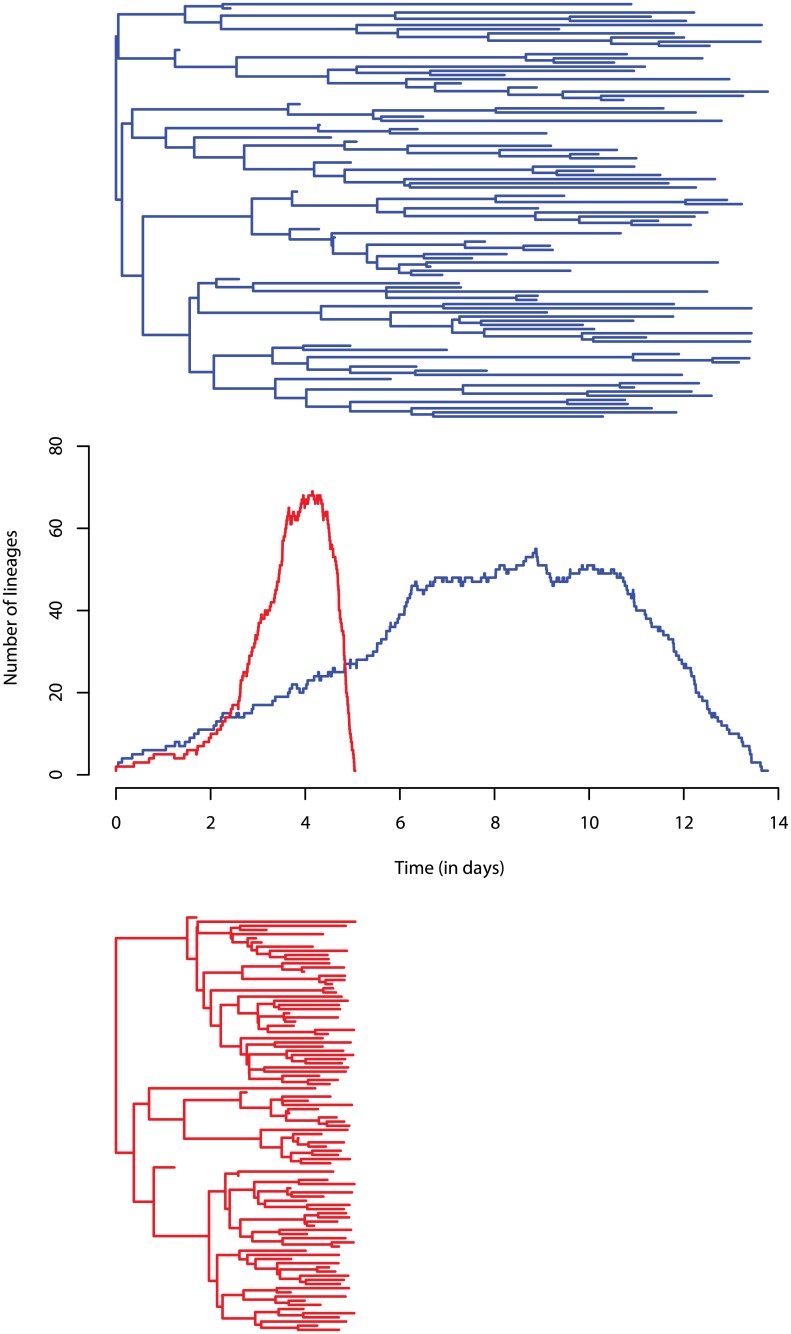
Simulated phylogenies of 100 leaves assuming a BD model and their corresponding LTT plot. The red phylogeny was simulated assuming *θ* = (*R*_0_ = 10, *d*_*I*_ = 5, *p* = 0.5) and the blue phylogeny was simulated assuming *θ* = (*R*_0_ = 2, *d*_*I*_ = 5, *p* = 0.5). Different *R*_0_s lead to different LTT plots and different tree shapes.

The Lineage-Through-Time (LTT) plot provides a graphical summary of a phylogeny [46]. It represents the number of lineages along the phylogeny as a piecewise constant function of time (Fig 2). Each step up in the LTT plot corresponds to a branching in the phylogeny, and each step down to a leaf. If all the infected individuals of an epidemics are sampled, the phylogeny corresponds to the full transmission tree and the LTT plot is identical to the prevalence curve. Therefore, as noted in earlier studies [22, 51–53], it is reasonable to presume that this plot could contain relevant information about epidemiological parameters. We summarized the LTT plot with two sets of summary statistics: one that captures particular metrics of the plot (Table 3) and another that simply uses the coordinates of its points as “summary” statistics. For this latter set of summary statistics, because the LTT plot contains as many points as there are nodes in the phylogeny (a phylogeny of *n* leaves has 2*n* − 1 nodes), and because here we consider phylogenies with more than 100 leaves, we averaged the points into 20 equally-sized bins, thus generating 40 summary statistics (20 x-axis coordinates and 20 y-axis coordinates).

To summarize, we used two main sets of summary statistics, the:

- sumstats set, with 43 summary statistics related to the tree and its LTT plot, which itself comprises three sets:
  - topo set: 8 topology summary statistics,
  - bl set: 26 branch-length summary statistics,
  - ltt set: 9 summary statistics related to the LTT plot,
- coords set, with 40 mean coordinates of the LTT plot.

Each summary statistic and all coordinates are computed recursively in O(n), where *n* is the number of leaves in the tree. This was a key criterion for the choice of the 83 statistics and is an important reason for the efficiency of our regression-ABC.

### Simulation study

We wanted to assess the potential of regression-ABC methods to infer epidemiological parameters from phylogenies. To this end, we first compared these methods to likelihood-based methods. We simulated “target” trees under several scenarios. In particular, we used the BD and the SIR epidemiological models to perform exhaustive comparisons. We expected our method to perform less well than likelihood-based methods since ABC, by definition, only approximates the likelihood function. However, practically speaking, the implementation of likelihood-based approach often requires simplifying assumptions to allow for efficient computation, which sometimes affects the results, as we show here.

We then compared a regression-ABC method to the kernel-ABC method presented by Poon [40], assuming the SI-DR model.

#### Target trees

For comparison with likelihood-based methods, we considered 32 scenarios, which correspond to all the combinations of:

- 2 epidemiological models (BD and SIR),
- 2 *R*_0_ values (*R*_0_ = 2, for a slow Influenza-like spread, and *R*_0_ = 10, for a rapid Measles-like spread),
- 2 durations of infection (*d*_*I*_ = 5 and *d*_*I*_ = 30),
- 2 sampling proportions (*p* = 0.05 and *p* = 0.5),
- 2 tree sizes (100 leaves and 1,000 leaves).

SIR target trees were all simulated in a population with *N* = 25,000 individuals. All simulations start at *t* = 0 in a population by the introduction of an infectious individual in a fully susceptible population of hosts and end when the number of samples is reached. This means we assume that the date at which the epidemic starts is known. For computational reasons, we limited the number of infected individuals to less than 3 ⋅ 10^5^, when assuming a BD model.

For comparison with the kernel-ABC method, we considered 8 scenarios, which correspond to all the combinations of:

- 2 contact rates associated with risk group 1 (*c*_1_ = 0.5 and *c*_1_ = 2),
- 2 tree sizes (300 leaves and 1,000 leaves),
- 2 types of trees (ultrametric and non-ultrametric).

We followed the protocol of the reference article [40] to simulate target trees within the rcolgem coalescent framework [23, 24] (S2 Text for details).

To perform a statistical performance analysis we simulated 100 target trees (replicates) for each of the scenarios.

#### Simulated “training” trees for regression-ABC

To train the regression-ABC, we simulated a set of 10,000 trees for each of the scenarios, using the same simulation system used to produce the target trees.

For comparison with likelihood-based methods on the BD and SIR models, we assumed the values of all the epidemiological parameters to be distributed in uniform priors (see Table 4). Again, for computational reasons, we imposed that the number of infected individuals through time remained lower than 3 ⋅ 10^5^ during simulation, when assuming a BD model.

**Table 4:** Prior table.

Parameter	Target value	Prior range
*R*_0_	2 10	U(1;5) U(5;20)
*d*_*I*_	5 30	U(1;15) U(7;60)
*N*	25,000	$U(10^{4};\;5\cdot 10^{4})$
*p*	0.05 0.5	U(0.01;0.1) U(0.4;0.6)

For the comparison with the kernel-ABC method, we used the same prior distributions as in [40] (S3 Text).

#### Correlation analysis

After simulating trees and computing the 83 summary statistics on every training tree, we calculated Spearman’s correlations between each of the summary statistics and epidemiological parameters to determine where the information was located in the trees.

#### Regression-ABC

We used the *abc* function from the abc R package [37, 54] to infer posterior distributions from rejection alone (ABC), and regression-ABC with feed-forward neural network (ABC-FFNN).

This function performs the rejection algorithm of Beaumont et al. [33] using a tolerance parameter *P*_*δ*_, which represents a percentile of the simulations that are close to the target. The proximity of the simulations to the target is evaluated in the function via the Euclidean distance between each normalized simulated vector of summary statistics, and the normalized target vector. The acceptance region is therefore spherical. The computation of the rejection step itself (once summary statistics are computed) is in O(TΣ), where *T* represents the size of the training set and *Σ* the number of summary statistics.

Prior to adjustment, the *abc* function performs smooth weighting using an Epanechnikov kernel as for the loc-linear adjustment proposed by Beaumont et al. [33]. We then performed an FFNN adjustment using the option available in the *abc* function [54]. This adjustment involves the construction of a non-linear conditional heteroscedastic regression model, using the *nnet* function (nnet R package), which involves an FFNN with a single-hidden-layer [37]. The *nnet* function includes a regularization of the fitting criterion through a penalty on “roughness”. This penalty, called “weight decay”, corresponds to the sum of the squares of the weights put on the links of the neural network and it contributes to avoiding over-fitting [55]. Bishop [56] also states that choosing a number of hidden units lower than the number of variables leads to dimensionality reduction and smoother regression. We used the default parametrization of the *abc* function, which does not provide perfect control over regularization and overfitting, and uses 5 FFNN hidden units.

In addition to simple rejection (ABC) and ABC-FFNN, we also used linear adjustment with variable selection using Least Absolute Shrinkage and Selection Operator (LASSO) regression [57]. The choice of such a regression model that performs well-controlled dimensionality reduction was motivated by the high number of summary statistics.

We implemented the LASSO adjustment (ABC-LASSO) using the glmnet R package [58]. As in the ABC-FFNN method, we weighted the simulations retained by rejection using an Epanechnikov kernel and we corrected for heteroscedasticity. LASSO performs variable selection naturally [57]. We optimized the number of selected variables using cross-validation with the *cv.glmnet* function. A multi-response Gaussian LASSO model was then computed using the *glmnet* function. The information regarding variable selection was kept to see whether some specific summary statistics are selected more often than others.

It is difficult to estimate the computational complexity of the regression-ABC approaches presented here because their algorithm involves four steps: first, the simulations; second, computation of summary statistics; third, rejection; and fourth, learning and regression. We know that the third and fourth steps are substantially less time-consuming than the first and second steps. The speed of the fourth step also depends on many variables: the size of the training set, the number of parameters to estimate, the number of summary statistics and, particularly, the machine learning technique being used. LASSO is presumed to run faster than FFNN (if the cost of cross-validation is not taken into account).

For completeness, we performed rejection using the distance between two LTT plots as a functional distance (ABC-D). We were inspired to do this by the function *nLTTstat* (nLTT R package), which computes the difference between two normalized LTT plots [59]. However, we did not normalize the LTT plots, to account for the potential temporal shift between two LTT plots (Fig 2).

In our comparisons, we ran these ABC methods to estimate the parameters of the target trees, using the sumstats and coords sets of summary statistics together or separately. We also used different tolerance proportions *P*_*δ*_ = {0.01; 0.05; 0.1; 0.2; 0.3; 0.4; 0.5} to determine the optimal value for each method.

#### Likelihood-based inference

We inferred the posterior distributions of the epidemiological parameters of the target trees using the likelihood-based approaches implemented in BEAST2 [27]. These methods are often used to infer the phylogeny and the epidemiological parameters from dated sequences simultaneously, but they also allow the user to assume that the phylogeny is known. In order to obtain comparable results, we ran BEAST2 with the same simulated time-scaled phylogenies as we used for ABC (see [38] for a similar methodology). We also used the same priors in BEAST2 and in our simulations to train ABC methods. The BEAST2 Markov chains were run for 10^6^ steps for all BD scenarios except the four scenarios with large trees and low sampling (1,000 leaves and *p* = 0.05), which required 5 ⋅ 10^6^ steps for convergence. For SIR scenarios, we ran chains of 10^7^ steps with 100-leaves trees, chains of 2 ⋅ 10^7^ steps with large trees, dense sampling and *R*_0_ = 2, chains of 5 ⋅ 10^7^ steps with large trees, dense sampling and *R*_0_ = 10, and chains of 10^8^ steps with with large trees and low sampling. For all BEAST2 posterior distributions (BEAST2-BD and BEAST2-BDSIR), we discarded the first 10% of the estimates as burn-in, and controlled for convergence using the Effective Sample Size measure (ESS) for the epidemiological parameters. We checked that ESS was greater than 200 for *R*_0_ and *d*_*I*_, and greater than 100 for *N*.

#### Kernel-ABC inference

The kernel-ABC approach is based on a functional distance, which measures topological dissimilarities between trees, weighted by the discordance in branch lengths. We reproduced the analysis with the kernel-ABC approach on the four sets of small target trees (300 leaves) presented above, using the same settings as [40] (S3 Text for more details about the kernel-ABC settings). For all kernel-ABC posterior distributions, we discarded the first 10% of the estimates as a burn-in.

#### Performance analysis

We measured the median ($\hat{\theta_{i}}$) and the 95% Highest Posterior Density (HPD_95%_) boundaries of each parameter posterior distribution (*D*_*i*_). For each ABC or BEAST2 run and each simulated scenario (100 target trees), we computed the mean relative error (MRE) as

$$\text{MRE}=\frac{1}{100}\sum_{i=1}^{100}\frac{1}{\theta }\left|\hat{\theta_{i}}-\theta \right|,$$

the mean relative bias (MRB) as

$$\text{MRB}=\frac{1}{100}\sum_{i=1}^{100}\frac{1}{\theta }\left(\hat{\theta_{i}}-\theta \right),$$

the mean relative 95% HPD width as

$${\text{width}}_{95\%}=\frac{1}{100}\sum_{i=1}^{100}\frac{1}{\theta }\left({\text{quantile}}_{97.5\%}\left(D_{i}\right)-{\text{quantile}}_{2.5\%}\left(D_{i}\right)\right)$$

and the 95% HPD accuracy as

$${\text{accuracy}}_{95\%}=\frac{1}{100}\sum_{i=1}^{100}1_{\left\{{\text{quantile}}_{2.5\%}\left(D_{i}\right)\leq \theta \leq {\text{quantile}}_{97.5\%}\left(D_{i}\right)\right\}}$$

We first tested the influence of the tolerance parameter on the mean relative error (MRE) of the four ABC algorithms (ABC, ABC-D, ABC-FFNN and ABC-LASSO). We then compared the performance of all these methods to that of likelihood-based methods implemented in BEAST2, assuming the same models and priors. We also compared the accuracy of our ABC-LASSO inferences to that of the kernel-ABC method, assuming the SI-DR model and using the same priors. Lastly, we tested the influence of the epidemiological parameter values used in each SIR scenario on the estimation error (MRE).

### Data analysis: The early stages of the 2014–2015 Ebola epidemic in Sierra Leone

Stadler et al. inferred epidemiological parameters using Ebola full-genome sequences from the 2014–2015 epidemic using BEAST2 and assuming the BDEI model (BEAST2-BDEI) [8]. Even though many more sequences have been released since then, this dataset remains interesting and relevant for comparing our regression-ABC to another likelihood-based approach. From an epidemiological standpoint, it remains one of the most densely sampled outbreaks in their early phase.

For this data analysis, Stadler et al. used 72 sequences obtained from patients in Sierra-Leone by Gire et al. [60]. We therefore used the RaxML phylogeny inferred by Gire et al. [60], which was computed on 81 sequences: 3 from Guinea patients and 78 from Sierra-Leone patients. We pruned all non-Sierra-Leone leaves. To compare our estimates with theirs, we followed their protocol by also pruning 6 leaves of the phylogeny corresponding to a sub-epidemics in Sierra-Leone. The remaining 72 sequences were sampled from late May to mid-June 2014. Using the known sampling dates, we scaled the phylogeny over time using the Least-Squares Dating (LSD) software, which uses fast algorithms and achieves accuracy comparable to more sophisticated methods [61].

We assumed a BDEI model and therefore estimated *R*_0_, *d*_*I*_ and the mean duration of latency *d*_*E*_, as in Stadler et al. [8]. As for previous models, the sampling proportion could not be estimated together with the other parameters due to identifiability problems [43].

The Ebola epidemic in Sierra Leone is thought to have started 6 months before it was officially identified and the first sample collected [8, 60]. Since our simulations start assuming the insertion of an infectious individual in a fully susceptible population of hosts, we therefore need to consider an additional simulation parameter, *origin*, which, in our simulations, corresponds to the time (in days) between the beginning of the epidemic in Sierra Leone and the beginning of sampling. Over this time period, the sampling rate was assumed to be *ε* = 0.

We simulated a set of 10,000 “training” trees assuming a BDEI model. For comparison purposes, we first used priors identical to those used in Stadler et al. for their BEAST2-BDEI inferences (see column *p* ≈ 0.7 in Table 5). We then used a different interval for the prior on the sampling proportion (*p* ≈ 0.4), because another study suggested that the sampling proportion lies between 0.2 and 0.7 [9]. Moreover, to simulate only biologically realistic epidemiological scenarios [62], we discarded all simulations where the total number of cases rose above 50,000 individuals.

**Table 5:** Prior table for Ebola data.

	Prior range
Parameter	Assumption *p* ≈ 0.7 [8] | *p* ≈ 0.4
*origin*	U(0,92)
*R*_0_	LN(0,1.25)
*d*_*E*_	Γ(0.5, 6)^−1^ ∈ [1; 26]
*d*_*I*_	Γ(0.5, 6)^−1^ ∈ [1; 26]
*p*	B(70,30) | B(25,35)

As in the simulation study, we computed Spearman’s correlation coefficients between each parameter of the set of simulated trees and the summary statistics.

Rejection is a determinant step in regression-ABC with adjustment because it selects the simulated data that will be used for learning. Even if the chosen regression model is robust, it can collapse if the rejection step fails to retain a relevant training set. The goodness-of-fit test implemented in the *gfit* function of the abc R package [54, 63] is an important preliminary test to be made in data analysis because it indicates whether the summary statistics are informative regarding target parameters. This test uses rejection based on the Euclidean distance on normalized entries, as defined by Beaumont et al. [33].

As dating of the Ebola phylogeny seemed poorly estimated (S1 Fig), we performed an upstream test of summary statistics goodness-of-fit of the “training” set against the phylogeny.

We inferred the posterior distributions of *d*_*E*_, *d*_*I*_ and *R*_0_ for the Ebola phylogeny using our ABC-LASSO regression model with *P*_*δ*_ = 0.5. We then compared our own estimates for the epidemiological parameters of the early spread of the Ebola epidemic in Sierra Leone with those obtained using the likelihood-based methods of Stadler et al [8]. Lastly, we analyzed the variables selected by the LASSO.

## Results

### Locating the epidemiological information in the phylogeny

**Fig 3:**
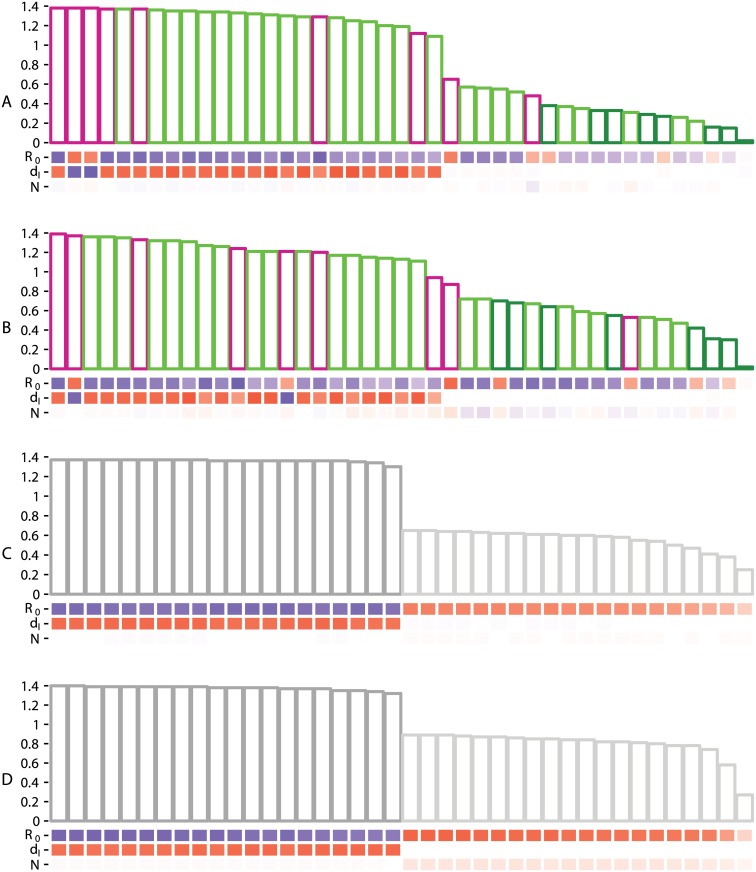
Heat map and histogram of Spearman’s correlations between the SIR model parameters and all sets of summary statistics for trees of 100 (A and C) or 1,000 (B and D) leaves. In panels A and B, the colors correspond to the bl (light green), topo (dark green) and ltt (magenta) sets. Panels C and D show the coords set related to the LTT plot with x-axis (dark gray) and y-axis (light gray) coordinates. Bar heights in the histograms represent the sum of the absolute correlations of each summary statistic to the whole set of parameters. Summary statistics and coordinates are ranked from the most to the least correlated. Correlation values between each summary statistic (or coordinate) and each epidemiological parameter are displayed in the heat map, where squares are colored with a gradient going from red (highly correlated positively) to white (no correlation) and blue (highly correlated negatively). The summary statistics names and correlations values for panels A, B, C and D, are given in S1, S2, S3 and S4 Tables respectively.

Fig 3 shows that the summary statistics computed on the Lineage-Through-Time plot (ltt set) are those that most correlate to the epidemiological parameters of the SIR model. The summary statistics describing the branch lengths (bl set) are less correlated and the topological summary statistics (topo set) are, in general, poorly correlated to the parameters. However, the topo set becomes more informative when the tree size increases, most likely because topological patterns become more distinguishable. There is little difference in the summary statistics histograms for trees of 100 leaves and trees of 1,000 leaves, the latter being more heavy tailed. bl set summary statistics are correlated positively to the duration of infection (*d*_*I*_) and correlated negatively to *R*_0_ (S1 and S2 Tables). None of the topological summary statistics are correlated to *d*_*I*_, even though they are correlated with *R*_0_. The coordinates of the LTT plot that are the most correlated to the epidemiological parameters are those of the x-axis, which are correlated positively to *d*_*I*_ and negatively to *R*_0_ (S3 and S4 Tables). Y-axis coordinates of the LTT plot strongly correlate positively with the *R*_0_ and weakly with the effective population size *N*.

Overall, *R*_0_ is the epidemiological parameter that is the most correlated to all summary statistics, which suggests that ABC approaches should be able to infer this parameter reliably. On the opposite, Fig 3 raises doubts about the ability of ABC approaches to infer the effective population size from phylogenies, because this parameter is poorly correlated to all of the summary statistics.

The correlations found for the BD model are very similar to those of the SIR model (S2 Fig and S5, S6, S7 and S8 Tables).

For the SI-DR model, which introduces host heterogeneity, the LTT plot summary statistics (ltt set) are correlated less strongly to the epidemiological parameters, whereas the y-axis coordinates of the LTT plot are correlated more strongly (S3 Fig, and S9, S10, S11 and S12 Tables). These y-axis coordinates are mostly correlated positively to *c*_1_ (contact rate associated with risk-group 1), *β* (transmission rate) and *N*, and negatively to *γ* (virulence). The summary statistics of the topo set are more correlated to the SI-DR parameters when trees are non-ultrametric than when they are ultrametric. However, even for this model, correlation remains low.

### Estimating the appropriate tolerance value

In this sub-section, we study the influence of the tolerance parameter used in the rejection step, on the inference error of our four ABC methods: standard rejection (ABC), rejection using the function distance between two LTT plots (ABC-D), rejection and adjustment using regularized neural networks (ABC-FFNN), and rejection and adjustment using LASSO (ABC-LASSO).

**Fig 4:**
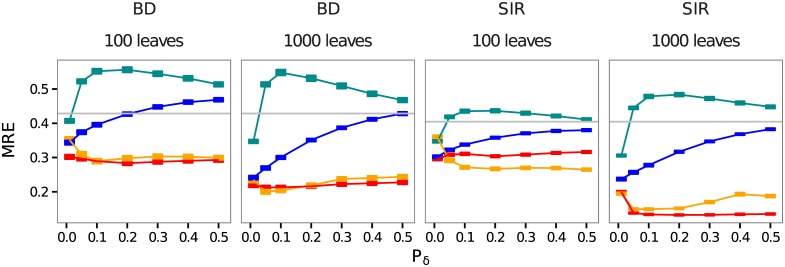
Influence of the tolerance parameter on the error for four ABC approaches used on all summary statistics. The x-axis shows the tolerance value. Squares represent the mean relative errors for each tolerance value with their standard errors. We show errors generated by ABC-D in turquoise, by ABC in blue, by ABC-FFNN in orange and by ABC-LASSO in red. The gray horizontal lines correspond to the mean relative error of the prior (i.e. expected error in rejection with a tolerance of 1). Results are displayed for both BD and SIR models and trees of both 100 leaves and 1,000 leaves.

We expected the errors of inference of ABC and ABC-D to increase with tolerance. Indeed, higher tolerance values should cause the rejection step to retain trees that are increasingly dissimilar to the target tree, that is, which have been generated by parameter values that are increasingly distant from the target values. Globally, this is what we observe in Fig 4. With large tolerance values, the error seems to converge towards that of the prior (the horizontal gray line), suggesting that there is not sufficient signal in the summary statistics to infer *d*_*I*_ by ABC and ABC-D.

Regarding the ABC-FFNN method, when the tolerance value increases, we expected the error to decrease at first (because the adjustment method used here requires a certain amount of training data) and finally to reach a plateau (when we have enough data and regularization can control for overfitting effects). This is the case for the inference of epidemiological parameters on small trees. For large trees, the error increases at the end for high tolerance values, which could be due to a poorly controlled regularization or to the limited size of the neural-network in the abc R function.

Concerning the ABC-LASSO method, we expected an increase in the tolerance value to decrease the inference error at first for the same reason as for the FFNN. However, in Fig 4, we only observe this effect for the SIR model with large trees. We then expected the error to reach a plateau and finally to increase because increasing the size of the training data increases the probability of non-linearity, which is problematic for the LASSO (linear) regression model. ABC-LASSO does not seem to reach the non-linearity zone in the tolerance range we considered here. The relative errors of the ABC-LASSO method remain below the threshold represented by the error induced by the prior (S5 Fig). Overall, the error with this approach is quite stable, likely due to well-controlled regularization.

We also analyzed the influence of the tolerance parameter on the 95% Highest Posterior Density (HPD) width (*width*_95%_). As expected, the posterior distributions obtained using regression-ABC methods are more adjusted than those obtained using the ABC-D or standard ABC method (S6 Fig). The *width*_95%_ of the posteriors obtained using ABC, ABC-D or ABC-FFNN increases with the tolerance, whereas that of the ABC-LASSO posteriors seems to be insensitive to tolerance parameter.

Overall, 0.01 is the best tolerance value for rejections without adjustment, and 0.5 is the best value with adjustment. Since this result was observed for both the BD and the SIR models, we adopted these values as default values for the remainder of the study.

### Comparison with likelihood-based approaches

Globally, BEAST2 achieved good convergence toward the epidemiological parameters posterior distributions, except for the large target trees simulated assuming the SIR model with *p* = 0.05 and *R*_0_ = 2. For those target trees, less than 20% of the *N* parameter posterior distributions had an ESS above 100.

**Fig 5:**
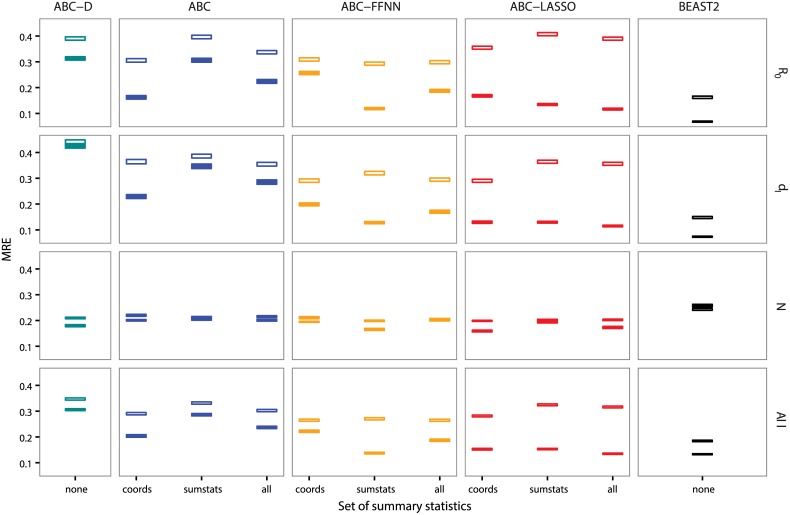
Inference errors on epidemiological parameters of SIR model using four ABC approaches with different sets of summary statistics. The x-axis shows the sets of summary statistics used. Squares represent mean errors with their standard errors. Empty squares correspond to results obtained on trees of 100 leaves and filled squares correspond to results on trees of 1,000 leaves. We show errors generated by ABC-D in turquoise, by ABC in blue, by ABC-FFNN in orange, by ABC-LASSO in red and by BEAST2-BDSIR in black. We show the average errors (bottom row) and the error for each parameter of interest.

Fig 5 shows that, for the SIR model and for large trees (1,000 leaves), regression-ABC methods can approach the accuracy of the likelihood-based approach (BEAST2-BDSIR, in black) and even outperform it for the inference of the effective population size. This can be explained by the fact that the BEAST2-BDSIR assumes an approximation of the true SIR model to speed up MCMC computations. Moreover, in the BDSIR model, the approximation of the number of susceptible individuals through time, *S*(*t*), potentially makes the effective population size *N* hard to estimate [42].

The standard ABC method (in blue) already provides good estimations of *R*_0_, consistently with Spearman’s correlations (Fig 3). We also find that the Euclidean distance between LTT plot coordinates (coords set, in blue) yields more accurate estimates than the functional distance between two LTT plots (ABC-D, in turquoise). This can be explained by the fact that in the functional distance we only consider the differences on the y-axis of the LTT plots, while in the standard ABC using the coords set we also consider the differences on the x-axis, which represents the time variable and are the most correlated to epidemiological parameters (Fig 3).

The performance of both regression-ABC methods is comparable when we consider small trees, and the accuracy of epidemiological parameter inference is always better for large trees. Note that, ABC-FFNN provides highly variable results for large trees, suggesting that regularization is poorly controlled in the algorithm we used.

ABC-LASSO always gives better estimations than the standard ABC on large trees. It also gives reliable results regardless of the set of summary statistics used. This suggests that our LASSO implementation is robust concerning the high number of explanatory variables. We analyzed which variables were selected in the LASSO regression models but we did not identify any strong selection pattern. This might be explained by the fact that many variables are highly correlated. It is also a known fact that variable selection using LASSO can be unstable [64].

Results concerning the BD model are presented in S7 Fig and are globally similar to observations for the SIR model, except that none of the ABC methods outperforms BEAST2-BD. This is consistent with the fact that BEAST2-BD is based on the exact likelihood function of the BD model. Nevertheless, the accuracy of ABC-LASSO on large trees is close to that of BEAST2-BD.

**Fig 6:**
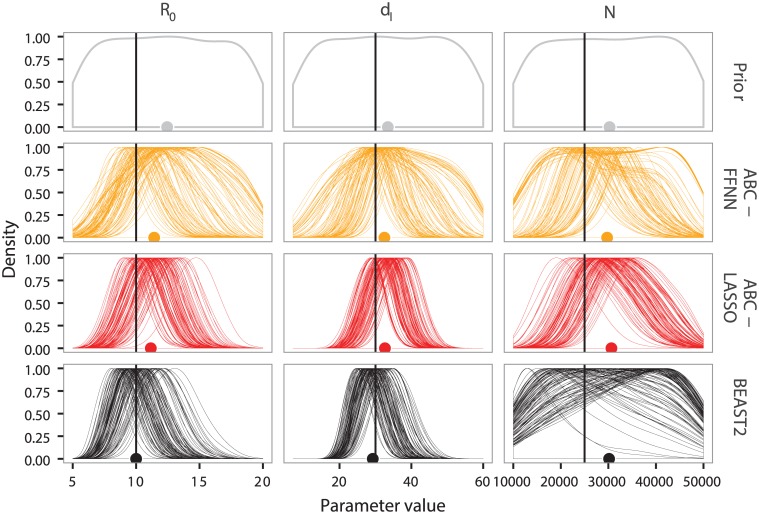
Prior and posterior distributions for parameter estimations by ABC-FFNN, ABC-LASSO and BEAST2-BDSIR. Prior distributions are in gray, posterior distributions obtained by ABC-FFNN are in orange, those by ABC-LASSO are in red and those by BEAST2-BDSIR are in black. All summary statistics were used for both regression-ABC approaches. We displayed the results for one particular epidemiological scenario (*R*_0_ = 10, *d*_*I*_ = 30 and *p* = 0.5) and for large trees. There are 100 replicates in this scenario. The dots represent the median of the posterior distribution merging for all replicates. The vertical black line represents the true value for each epidemiological parameter.

Fig 6 gives the example of a particular SIR scenario (dense sampling, high *R*_0_, and high *d*_*I*_), where for large time-scaled phylogenies (Fig 6B), the majority of the replicates of ABC-LASSO converge towards a posterior distribution, which is adjusted and centered approximately on the target value. This is also true for the BD model (S8 Fig). We find similar posterior distributions for the likelihood-based approach except for the *N* parameter, where the posterior clearly reveals a lack of convergence.

### Results for the SI-DR model

We only ran ABC-LASSO using the sumstats and coords sets of summary statistics together, and set *P*_*δ*_ to 0.5. As shown in Table 6, for non-ultrametric target trees simulated with *c*_1_ = 2, ABC-LASSO infers *c*_1_ very accurately (*MRE* = 0.065). Inferring *β* with this method is slightly more difficult (*MRE* = 0.24), but the target value of *β* always falls into the 95% Highest Posterior Density (*accuracy*_95%_ = 100). Unfortunately, we fail to infer *γ* and *N*. However both parameters are easier to infer when *c*_1_ = 2 than when *c*_1_ = 0.5. As shown in Table 6, with ABC-LASSO, all four parameters of the SI-DR model, especially *N* and *γ*, are better inferred from large trees (${\bar{\text{MRE}}}_{1000}=1.14$ whereas ${\bar{\text{MRE}}}_{300}=8.09$). We also observe an effect of the ultrametric nature or not of the target trees. Unlike other parameters, the inference error on *β* is lower with non-ultrametric trees than with ultrametric trees. Despite these contrasted results, ABC-LASSO outperforms the kernel-ABC method from [40] for all parameters. This is not affected by increasing the length of the MCMC chain to 50,000 steps for kernel-ABC.

**Table 6:** Performance of the ABC-LASSO and the kernel-ABC methods on non-ultrametric trees (*c*_1_ = 2). Mean Relative Error (MRE) and 95% HPD accuracy (*accuracy*_95%_) of inference of the SI-DR epidemiological parameters by both ABC-LASSO and kernel-ABC approaches. For the ABC-LASSO method, we show the results obtained on the 100 large target trees (1,000 leaves) enclosed in brackets. For the kernel-ABC method, we show the results obtained after extending the MCMC chain length to 50,000 steps for 10 target trees enclosed in square brackets.

parameter	method	MRE	accuracy_95%_
*β*	ABC-LASSO kernel-ABC	0.24 (0.39) 20 [11]	100 (100) 3 [0]
*c*_1_	ABC-LASSO kernel-ABC	0.065 (0.055) 0.44 [0.41]	100 (100) 6 [0]
*γ*	ABC-LASSO kernel-ABC	2 (1.4) 9.3 [4.9]	7 (56) 8 [3]
*N*	ABC-LASSO kernel-ABC	2 (1.5) 2.9 [3.2]	22 (75) 4 [3]

We ran additional analyses to compare the kernel distance with the our summary statistics using a simple rejection (S4 Text). Results indicated that the kernel distance is less correlated to the inference task than the Euclidean distance computed from all of our summary statistics together (S9 Fig).

### Ebola phylodynamics

We analyzed the correlation between the epidemiological parameters of the BDEI model and the summary statistics or coordinates of the LTT plot for trees of 72 leaves (S4 Fig). As previously observed for the SIR model, we see that the summary statistics computed on the Lineage-Through-Time plot (ltt set) and those computed on the branch lengths (bl set) are the most correlated to the epidemiological parameters. Conversely, the topological indexes (topo set) contain very little information about the parameters. The bl summary statistics are correlated positively to both the duration of infectiousness *d*_*I*_ and the duration of latency *d*_*E*_, except the *ie_BL_median_[1]* statistics, which is correlated negatively to *d*_*E*_ and correlated positively to *d*_*I*_ (S13 Table). The coordinates of the LTT plot (coords set) are correlated poorly to *d*_*E*_ (S14 Table).

As for any data analysis, it is important to assess the fitness of the summary statistics to infer the epidemiological parameters from the “target” phylogeny. We did this for the sumstats and coords sets together and separately. The goodness-of-fit test revealed that the coords set of summary statistics was not fit to infer the epidemiological parameters of the Ebola phylogeny (p-value < 0.05). Therefore we only used the sumstats set of summary statistics.

**Fig 7:**
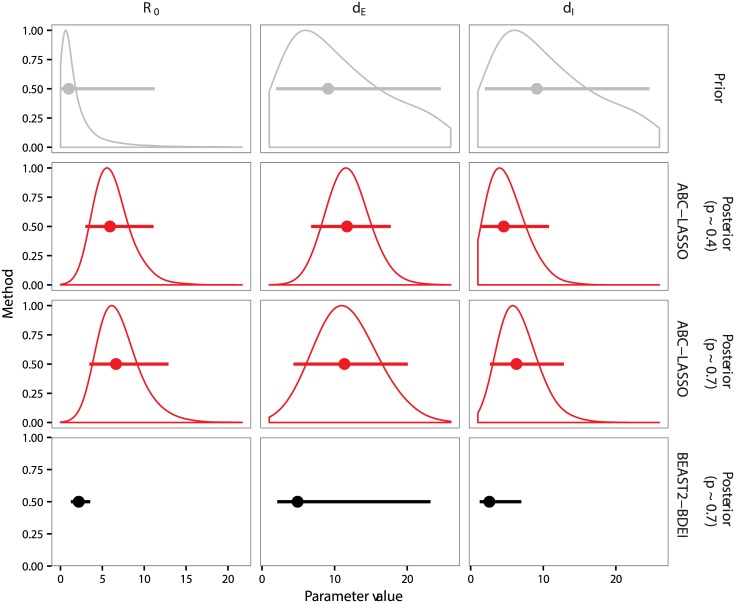
Prior and posterior distributions of parameter estimations from the Ebola phylogeny. We show the results for two different inference methods: ABC-LASSO (in red) and BEAST2-BDEI (in black). Gray distributions correspond to the prior and red distributions correspond to ABC-LASSO posterior distributions. The dots represent the median and the vertical lines represent the 95% highest posterior density of each distribution. Statistics on the BEAST2-BDEI posterior distributions were obtained from [8].

Fig 7 shows that the median of the posterior distribution of *R*_0_, inferred by Stadler et al. using BEAST2-BDEI, is close to the median of their prior distribution (in gray). The duration of latency seems very difficult to infer using the BEAST2 approach, as *d*_*E*_ HPD 95% is almost as large as that of the prior.

Our parameter estimates differ slightly from those of Stadler et al. We find a longer incubation period (11.7 [HPD_95%_: 6.77–17.74]) and a longer duration of infectiousness (4.5 [HPD_95%_: 1.41–10.79]) than Stadler et al (4.92 [HPD_95%_: 2.11–23.20] and 2.58 [HPD_95%_: 1.24–6.98] respectively). Both of these are more in line with the estimations from the WHO Ebola Response Team [65], which found that the fitted incubation period was 9.9 ± 5.6 days and the mean duration of infectiousness in the community was about 4.6 ± 5.1 days. We also infer a greater value for *R*_0_ than Stadler et al (5.92 [HPD_95%_: 2.97–11.12] instead of 2.18 [HPD_95%_: 1.24–3.55]), which is probably driven by the longer duration of latency. Indeed, even if the duration of latency does not appear in the deterministic formulation of *R*_0_ for the BDEI model, it may have an effect in the stochastic setting. Put differently, we have more infected individuals in our simulations, but a high proportion of these individuals are still latent and do not propagate the disease. Our *R*_0_ estimation is more in line with [9], which used the same dataset but fixed the duration of latency, and found that *R*_0_ = 2.40 [HPD_95%_: 1.54–3.87] if *d*_*E*_ = 5.3 days and *R*_0_ = 3.81 [HPD_95%_: 2.47–6.3] if *d*_*E*_ = 12.7 days.

As the phylogeny from [60] that we used in this study is poorly supported (average bootstrap support = 0.23), we performed a supplementary analysis to assess the robustness of our method in the presence of phylogenetic uncertainty (S5 Text). We used 10 additional trees with nearly optimal likelihood scores, and showed that, despite the presence of substantial topological differences (average normalized RF distance among trees equal to 0.23 [66]), the posterior distributions inferred by ABC-LASSO are very similar (S10 Fig).

## Discussion

Extracting epidemiological information from pathogen phylogenies largely remains an open challenge, especially for large phylogenies and complex models [12]. Here, we show that regression-based Approximate Bayesian Computation (ABC) involving a large number of summary statistics to describe the phylogeny offers a promising alternative to existing methods.

### Summary statistics

For the BD and the SIR models, we found that the shape of the phylogeny contained less information about the epidemiological parameters than the LTT plot and the branch lengths. We also did not find any strong correlation between topological statistics and epidemiological parameters for the SI-DR model, which captures host structure and therefore could be expected to make these statistics more relevant [39, 40, 67, 68]. However, we found the lineage component (y-axis) of the LTT plot, which is related to the topology, to be more correlated to the epidemiological parameters in the SI-DR model than in all the other models we studied. Our current set of summary statistics seems to be sufficient to infer the epidemiological parameters of the BD and the SIR models, but not those of the SI-DR model. In fact, our results on this model show that our summary statistics are quite poorly correlated to the two epidemiological parameters that we have difficulties to infer (infection duration and population size). This suggests that there is no universal set of summary statistics and that there is room for additional ones, to be used to analyze the SI-DR model and likely other complex models.

### Regression-ABC

Summary statistics are sometimes viewed as the Achilles’ heel of ABC, because “summarizing” suggests a loss of information. Furthermore, complex objects such as phylogenies can contain information unrelated to epidemiological parameters, which may dilute the desired signal. Selecting the “relevant” summary statistics could improve the method’s accuracy, but this is notoriously difficult [39, 69–71]. Here, we show that current machine learning techniques are efficient at performing variable selection on a large number of summary statistics.

One potential limitation of the rejection approach we used is that it relies on the simple Euclidean distance between unweighted summary statistics. One option could be to use adaptive methods of distance weighting, but these are time consuming and tend to be replaced by machine learning techniques.

The comparison between the LASSO and FFNN regression methods revealed that ABC-LASSO was more robust to the choice of summary statistics than ABC-FFNN. This was likely due to the R packages we used, and we expect that re-implementating an FFNN model with regularization tuning would remove this difference. The non-linearity of FFNN could then become an advantage. In theory, an advantage of LASSO compared to FFNN is that it provides us with an output on the selected summary statistics. However, we were unable to identify sets of summary statistics that were always selected or always discarded. This is likely due to the high degree of correlation between our summary statistics. A random forest approach could possibly circumvent these difficulties [72].

### Method comparison

We compared regression-ABC methods to the kernel-ABC method [40] and to likelihood-based approaches based on birth-death-sampling (BDS) processes [21, 22, 42]. Our choice was motivated by the fact that the former relies on ABC and that the latter is widely used thanks to BEAST2. Another powerful method, which is also likelihood-based, involves coalescent processes [19, 23]. We did not use this method for parameter inference because, to the best of our knowledge, it is currently only implemented in R and we anticipated issues with computing time. However, we did use the tree simulator (rcolgem) associated with this method for comparison with kernel-ABC.

In short, when comparing our ABC-LASSO method to the BDS methods, we obtained comparable (but slightly lower) accuracy when estimating *R*_0_ and infection duration. We also found that the accuracy of our ABC method always increases with phylogeny size. When assuming an SIR model, we obtained more accurate estimates of host population size than the BEAST2-BDSIR approach. The SI-DR epidemiological model is the model where the accuracy of the estimates using ABC-LASSO was globally the most disappointing (even though it was still better than with kernel-ABC). This could be due to the fact that we made several assumptions in order to compare our results to [40]. For instance, the tree size was relatively small (300 leaves) and our results showed that accuracy is better on larger trees (1000 leaves). It could also be that the target values chosen for the contact rates of the two host classes were too close (*c*_1_ = 0.5 or 2, and *c*_2_ = 1) to be well differentiated. The SI-DR model is a complex epidemiological model with many parameters and the four chosen by [40] are perhaps not all identifiable, at least when using our current set of summary statistics. It may be that developing additional summary statistics or using larger training sets to learn the regression model could improve the approach’s accuracy.

When comparing methods, we saw that posterior distributions can be much more valuable than statistics such as the relative error. Indeed, if the prior distribution is centered approximately on the targeted value, without any selection on parameter values the posterior will not deviate from it. This is illustrated, for instance, by the population size parameter in the SIR model, where some models have reasonable relative error even though the posterior is often identical to the prior (Fig 6).

Our results are consistent with those reported recently by the PANGEA-HIV consortium [28]. One aspect that deserves more investigation is related to computing time. Indeed, the most successful method in PANGEA-HIV required “considerable resources” in terms of CPU. The most time-consuming part in our ABC-LASSO is the simulation and the computation of the training set summary statistics. Rejection in itself is very fast, and LASSO is a fast machine-learning technique even if it is combined with cross-validation to avoid over-fitting. The computational complexity of simulation is generally linear with respect to the number of samples and the number of time-steps (or events) considered during the simulation. Moreover, the approach’s complexity also depends on the number and type of summary statistics. We chose to use a large number of summary statistics, but each of these is computed quickly in time at most linear in the tree size. Furthermore, the simulations and computation of summary statistics can both be run easily in parallel. In the likelihood-based methods we used, computing time depends on calculation of the likelihood function (which can be easy for the simple BD model and most coalescent models, but can be complicated due to the necessity to integrate over time for some others [22]) and on the convergence towards a posterior distribution (which is generally led by an MCMC search). Lastly, for the kernel-ABC approach, the computational complexity depends on that of the simulation procedure, the functional distance (which is much longer to compute than our simple Euclidean distance) and the MCMC search (which depends on the length of the MCMC chain and on the number of epidemiological parameters). This list suggests that regression-ABC may become advantageous when the number of training trees to learn the regression model becomes smaller than the length of the MCMC chain required to obtain convergence. Further investigation is warranted on this topic since both of these mehtods depend on the number of parameters to estimate, the size of the phylogenies, and also the relevance and information content of summary statistics.

### Perspectives

Our goal was to compare existing methods to determine whether regression-ABC can be an alternative to MCMC-based methods. We showed that this approach can reach an accuracy comparable to state-of-the-art techniques, which allows us to envisage several paths for future studies.

A direct extension of our approach could be to investigate more complex models, since the major requirement of our approach is to be able to rapidly simulate data assuming such models. Additional efforts will likely be needed to design new relevant summary statistics.

Another possibility would be to modify the method in order to take into account surveillance data [73] or to directly analyze sequence data. This latter modification would be valuable when the inference of a time-scaled phylogeny is difficult or impossible [12]. We could also include natural selection in the model to allow pathogen strains to spread at different speeds.

On the technical side, a promising extension would be to explore random forest algorithms, which are powerful tools for clustering and non-linear regression with high explanatory power [72]. These algorithms have already led to promising results in the ABC framework [74]. Lastly, we focused here on phylogenies of epidemics but this method could be extended to infer parameters from phylogenies generated using ecological or evolutionary models [75, 76].

## Supporting information

S1 Text Ordinary differential equation systems of BD, SIR and BDEI models. (PDF)

S2 Text Protocol for simulating target trees assuming the differential-risk model with the rcolgem coalescent framework. (PDF)

S3 Text Kernel-ABC settings. (PDF)

S4 Text Comparison between kernel distance and summary statistics distance. (PDF)

S5 Text ABC-LASSO robustness analysis. (PDF)

S1 Fig Ebola phylogenies and LTT plot. Panel A shows the pruned Ebola phylogeny, panel B shows the time-scaled Ebola phylogeny obtained by LSD and panel C shows the LTT plot corresponding to the time-scaled phylogeny.
(EPS)

S2 Fig Heat map and histogram of Spearman’s correlations between the epidemiological parameters of the BD model and all sets of summary statistics for trees of 100 (A and C) or 1,000 (B and D) leaves. In panels A and B, the colors correspond to the bl (light green), topo (dark green) and ltt (magenta) sets. Panels C and D show the coords set related to the LTT plot with x-axis (dark gray) and y-axis (light gray) coordinates. Bar heights in the histograms represent the sum of the absolute correlations of each summary statistic to the whole set of parameters. Summary statistics and coordinates are ranked from the most to the least correlated. Correlation values between each summary statistic (or coordinate) and each epidemiological parameter are displayed in the heat map, where squares are colored with a gradient from red (highly correlated positively) to white (no correlation) and blue (highly correlated negatively). The names of the summary statistics and the correlations values corresponding to panels A, B, C and D, are given in S5, S6, S7 and S8 Tables respectively.
(EPS)

S3 Fig Heat map and histogram of Spearman’s correlations between the epidemiological parameters of the SI-DR model and all sets of summary statistics for ultrametric trees (A and C) or non-ultrametric trees (B and D). In panels A and B, the colors correspond to the bl (light green), topo (dark green) and ltt (magenta) sets. Panels C and D show the coords set related to the LTT plot with x-axis (dark gray) and y-axis (light gray) coordinates. Bar heights in the histograms represent the sum of the absolute correlations of each summary statistic to the whole set of parameters. Summary statistics and coordinates are ranked from the most to the least correlated. Correlation values between each summary statistic (or coordinate) and each epidemiological parameter are displayed in the heat map, where squares are colored according to a gradient from red (highly correlated positively) to white (no correlation) and blue (highly correlated negatively). The names of the summary statistics and the correlations values corresponding to panels A, B, C and D, are given in S9, S10, S11 and S12 Tables respectively.
(EPS)

S4 Fig Heat map and histograms of Spearman’s correlations between epidemiological parameters of the BDEI model and all sets of summary statistics for trees of 72 leaves simulated assuming p ≈ 0.4. In panel A, the colors correspond to the bl (light green), topo (dark green) and ltt (magenta) sets. Panel B show the coords set related to the LTT plot with x-axis (dark gray) and y-axis (light gray) coordinates. On the x-axis, summary statistics or coordinates are ranked from the most to the least correlated to all epidemiological parameters. Bar heights in the histograms represent the mean absolute correlation of each summary statistic to the whole set of parameters. Summary statistics and coordinates are ranked from the most to the least correlated. Correlation values between each summary statistic (or coordinate) and each epidemiological parameter are displayed in the heat map, where squares are colored according to a gradient from red (highly correlated positively) to white (no correlation) and blue (highly correlated negatively). The names of the summary statistics and the correlations values corresponding to panels A and B are given in S13 and S14 Tables respectively.
(EPS)

S5 Fig Influence of the tolerance parameter on the MRE for four ABC approaches used on all summary statistics. The x-axis shows the tolerance value. Squares represent the MRE for each tolerance value with their standard errors. We show MRE generated by ABC-D in turquoise, by ABC in blue, by ABC-FFNN in orange and by ABC-LASSO in red. The gray horizontal lines correspond to the prior the MRE of the prior (i.e. expected error in rejection with a tolerance of 1). Results are displayed for both BD and SIR models, trees of both 100 leaves and 1,000 leaves and for all epidemiological parameters of interest (*R*_0_, *d*_*I*_ and *N*).
(EPS)

S6 Fig Influence of the tolerance parameter on the width_95%_ of the posterior distributions for four ABC approaches used on all summary statistics. The x-axis shows the tolerance value. Squares represent the mean width_95%_ for each tolerance value with their standard errors. We show width_95%_ corresponding to ABC-D in turquoise, to ABC in blue, to ABC-FFNN in orange and to ABC-LASSO in red. The gray horizontal lines correspond to the prior width_95%_. Results are displayed for both BD and SIR model and both trees of 100 leaves and 1,000 leaves.
(EPS)

S7 Fig Inference errors on epidemiological parameters of the BD model using ABC approaches with different sets of summary statistics. The x-axis shows the sets of summary statistics used. Squares represent mean errors with their standard errors. Transparent squares correspond to results obtained on trees of 100 leaves and opaque squares correspond to results on trees of 1,000 leaves. We show errors generated by ABC-D in turquoise, by ABC in blue, by ABC-FFNN in orange, by ABC-LASSO in red and by BEAST2-BD in black. We show the average errors (bottom row) and the error for each parameter of interest.
(EPS)

S8 Fig Prior and posterior distributions for parameter estimations by ABC-FFNN, ABC-LASSO and BEAST2-BD. Prior distributions are in gray, posterior distributions obtained by ABC-FFNN are in orange, those by ABC-LASSO are in red and those by BEAST2-BD are in black. All summary statistics were used for both regression-ABC approaches. We displayed the results for one particular epidemiological scenario (*R*_0_ = 10, *d*_*I*_ = 30 and *p* = 0.5) and for large trees. There are 100 replicates in this scenario. The dots represent the median of the merging of the posterior distributions for all replicates. The vertical black line represents the true value for each epidemiological parameter.
(EPS)

S9 Fig Comparison of the accuracy of ABC approaches based either on the kernel distance of [40] or on the summary statistics. The x-axis shows the tolerance value. We show the Mean Relative Error (MRE) corresponding to rejection using the kernel distance of [40] in green, to kernel-ABC in black and to ABC and ABC-LASSO based on all sets of summary statistics in blue and in red respectively. The gray lines correspond to the prior MRE for each parameter and each scenario (*c*_1_ = 0.5 or *c*_1_ = 2). Results are displayed for both ultrametric and non-ultrametric trees of 300 leaves simulated assuming the SI-DR model with *c*_1_ = 0.5 or *c*_1_ = 2.
(EPS)

S10 Fig Variations in posterior distribution estimated by ABC-LASSO from different inferred phylogenies. The dots represent the median and the vertical lines represent the 95% highest posterior density of each distribution. Gray distributions correspond to the prior and red distributions correspond to ABC-LASSO posterior distributions. The different ABC-LASSO posterior distributions were computed from the best RAxML phylogeny published by [60] and from the 10 best RAxML phylogenies (labelled from 1 to 10) inferred from the same sequence data set and using the same parameters as in [60] but from different random starting tree topologies.
(EPS)

S1 Table Table of correlations between the summary statistics of the bl, ltt and topo sets and the epidemiological parameters of the SIR model, for trees of 100 leaves. (PDF)

S2 Table Table of correlations between the summary statistics of the bl, topo and ltt sets and the epidemiological parameters of the SIR model, for trees of 1,000 leaves. (PDF)

S3 Table Table of correlations between the summary statistics of the coords set and the epidemiological parameters of the SIR model, for trees of 100 leaves. (PDF)

S4 Table Table of correlations between the summary statistics of the coords set and the epidemiological parameters of the SIR model, for trees of 1,000 leaves. (PDF)

S5 Table Table of correlations between the summary statistics of the bl, topo and ltt sets and the epidemiological parameters of the BD model, for trees of 100 leaves. (PDF)

S6 Table Table of correlations between the summary statistics of the bl, topo and ltt sets and the epidemiological parameters of the BD model, for trees of 1,000 leaves. (PDF)

S7 Table Table of correlations between the summary statistics of the coords set and the epidemiological parameters of the BD model, for trees of 100 leaves. (PDF)

S8 Table Table of correlations between the summary statistics of the coords set and the epidemiological parameters of the BD model, for trees of 1,000 leaves. (PDF)

S9 Table Table of correlations between the summary statistics of the bl, topo and ltt sets and the epidemiological parameters of the SI-DR model, for ultrametric trees of 300 leaves. (PDF)

S10 Table Table of correlations between the summary statistics of the bl, topo and ltt sets and the epidemiological parameters of the SI-DR model, for non-ultrametric trees of 300 leaves. (PDF)

S11 Table Table of correlations between the summary statistics of the coords set and the epidemiological parameters of the SI-DR model, for ultrametric trees of 300 leaves. (PDF)

S12 Table Table of correlations between the summary statistics of the coords set and the epidemiological parameters of the SI-DR model, for non-ultrametric trees of 300 leaves. (PDF)

S13 Table Table of correlations between the summary statistics of the bl, ltt and topo sets and the epidemiological parameters of the BDEI model, for trees of 72 leaves simulated assuming p ≈ 0.4. (PDF)

S14 Table Table of correlations between the summary statistics of the coords set and the epidemiological parameters of the BDEI model, for trees of 72 leaves simulated assuming p ≈ 0.4. (PDF)
