# Supplementary material for: Inferring epidemiological parameters from phylogenies using regression-ABC: A comparative study
Source: PLoS Comput Biol. 2017 Mar 6;13(3):e1005416. doi: 10.1371/journal.pcbi.1005416 (PMC5358897; doi:10.1371/journal.pcbi.1005416)
Supplement: S1 Text — (PDF) [file pcbi.1005416.s001.pdf]

S1 Text

Ordinary differential equation systems of BD, SIR and BDEI models.

In the following ordinary differential equation systems of BD (Eq. S1), SIR (Eq. S2) and BDEI (Eq. S3) models, $S(t)$, $E(t)$, $I(t)$ and $R(t)$ respectively denote the number of Susceptible, Exposed, Infectious and Removed individuals at time t.

$$\frac{\mathrm{d}I(t)}{\mathrm{d}t} = \beta I(t) - (\gamma + \varepsilon) I(t) \tag{S1}$$

$$\frac{\mathrm{d}S(t)}{\mathrm{d}t} = -\beta \frac{S(t)}{N} I(t) \tag{S2a}$$

$$\frac{\mathrm{d}I(t)}{\mathrm{d}t} = \beta \frac{S(t)}{N} I(t) - (\gamma + \varepsilon) I(t) \tag{S2b}$$

$$\frac{\mathrm{d}R(t)}{\mathrm{d}t} = (\gamma + \varepsilon) I(t) \tag{S2c}$$

$$\frac{\mathrm{d}E(t)}{\mathrm{d}t} = \beta I(t) - \sigma E(t) \tag{S3a}$$

$$\frac{\mathrm{d}I(t)}{\mathrm{d}t} = \sigma E(t) - (\gamma + \varepsilon) I(t) \tag{S3b}$$
