# Supplementary material for: Inferring epidemiological parameters from phylogenies using regression-ABC: A comparative study
Source: PLoS Comput Biol. 2017 Mar 6;13(3):e1005416. doi: 10.1371/journal.pcbi.1005416 (PMC5358897; doi:10.1371/journal.pcbi.1005416)
Supplement: S2 Text — (PDF) [file pcbi.1005416.s002.pdf]

S2 Text

Protocol for simulating target trees assuming the differential-risk model with the `rcolgem` coalescent framework.

Using a modified version of the `simulate.DiffRisk.R` script, which we found in the `kamphir-master/drivers/` directory of the online repository, we simulated four sets of target trees with the `rcolgem` coalescent framework [1, 2]: ultrametric trees of 300 leaves, non-ultrametric trees of 300 leaves, ultrametric trees of $1,000$ leaves and non-ultrametric trees of $1,000$ leaves.

For each set, we simulated two subset of 100 target trees assuming the following parameter values :

- $\beta = 0.01$ (transmission rate)
- $\gamma = \frac{1}{520}$ (additional mortality rate, i.e. virulence)
- $N = 3000$ (total population size)
- $\mu = \frac{1}{3640}$ (basal mortality rate)
- $c_2 = 1.0$ (contact rate associated with risk group 2)
- $\rho = 0.9$ (proportion of assortative mixing)
- $f = 0.5$ (frequency of risk group 1)

The first subset was simulated assuming $c_1 = 0.5$ and the second with $c_1 = 2$ (contact rate associated with risk group 1). These parameter values are identical to those used to validate the kernel-ABC method assuming the SI-DR model in [3].

As in [3], we used the following starting and stopping conditions for the simulations:

- $t_{end} = 30 \times 52$ (time between the beginning of the epidemic and the last sample)
- $ntips = 300$ or 1000 depending on the target set (sample size)
- $S_1 = f \times N - 1$ (initial number of susceptible individuals in risk group 1)
- $S_2 = (1 - f) \times N$ (initial number of susceptible individuals in risk group 2)
- $I_1 = 1$ (initial number of infectious individuals in risk group 1)
- $I_2 = 0$ (initial number of infectious individuals in risk group 2)

Sampling dates of ultrametric trees were all fixed to t_{end}. For non-ultrametric trees, we randomly drew $ntips$ (300 or $1,000$) sampling dates from a uniform law $\mathcal{U}(\frac{t_{end}}{2}; t_{end})$ (so tip heights are in $[0; \frac{t_{end}}{2}]$) and used these dates for all target trees (respectively trees of 300 or $1,000$ leaves).

In [3], the target trees were first simulated with the `rcolgem` coalescent framework then re-estimated using phylogenetic methods via sequence simulation. This was done "to provide idealized conditions for parameter estimation using either BEAST2 or the kernel-ABC method—in other words, to identify biases inherent to either framework rather than due to uncertainty in phylogenetic reconstruction" [3]. Here, we did not re-estimate the target trees. Thus we estimated parameter values directly from `rcolgem` trees. If anything, we expect this change to improve the performance of the kernel-ABC method.

References

[1] Volz EM. Complex population dynamics and the coalescent under neutrality. Genetics. 2012 Jan;190(1):187–201. Available from: `http://dx.doi.org/10.1534/genetics.111.134627`.

[2] Rasmussen DA, Volz EM, Koelle K. Phylodynamic inference for structured epidemiological models. PLoS Comput Biol. 2014 Apr;10(4):e1003570. Available from: `http://dx.doi.org/10.1371/journal.pcbi.1003570`.

[3] Poon AFY. Phylodynamic Inference with Kernel ABC and Its Application to HIV Epidemiology. Mol Biol Evol. 2015 Sep;32(9):2483–2495. Available from: `http://dx.doi.org/10.1093/molbev/msv123`.
