# Supplementary material for: Inferring epidemiological parameters from phylogenies using regression-ABC: A comparative study
Source: PLoS Comput Biol. 2017 Mar 6;13(3):e1005416. doi: 10.1371/journal.pcbi.1005416 (PMC5358897; doi:10.1371/journal.pcbi.1005416)
Supplement: S3 Text — (PDF) [file pcbi.1005416.s003.pdf]

S3 Text

Kernel-ABC settings.

For the kernel-ABC, the prior distributions of the four parameters of interest were defined as in the `example.DiffRisk.json` file provided by the kamphir-master software package (in the `kamphir-master/settings/` directory), all remaining parameters were fixed to their true value.

For our ABC-LASSO, we used exactly the same prior distributions and the same starting and stopping conditions (the latter being defined earlier) as for kernel-ABC, to simulate 10,000 "training" trees with the `rcolgem` coalescent framework [1, 2], for each set of target trees.

As defined in [3], we used the following kernel-ABC settings for the differential-risk model :

- $\lambda = 0.3$ (constant decay factor)
- $\sigma = 2.0$ (Gaussian radial basis function tolerance parameter)
- $\tau_0 = 0.005$ (initial tolerance)
- $\tau_{min} = 0.002$ (minimum tolerance)
- $\lambda_\tau = 0.0025$ (exponential decay rate)
- $10,000$ MCMC steps

We used a modified version of the kernel-ABC software to infer the four epidemiological parameters of the SI-DR model using this approach. This version is freely accessible from `http://dx.doi.org/10.6084/m9.figshare.4235354`.

References

[1] Volz EM. Complex population dynamics and the coalescent under neutrality. Genetics. 2012 Jan;190(1):187–201. Available from: http://dx.doi.org/10.1534/genetics.111.134627.

[2] Rasmussen DA, Volz EM, Koelle K. Phylodynamic inference for structured epidemiological models. PLoS Comput Biol. 2014 Apr;10(4):e1003570. Available from: http://dx.doi.org/10.1371/journal.pcbi.1003570.

[3] Poon AFY. Phylodynamic Inference with Kernel ABC and Its Application to HIV Epidemiology. Mol Biol Evol. 2015 Sep;32(9):2483–2495. Available from: http://dx.doi.org/10.1093/molbev/msv123.
