# Supplementary material for: Inferring epidemiological parameters from phylogenies using regression-ABC: A comparative study
Source: PLoS Comput Biol. 2017 Mar 6;13(3):e1005416. doi: 10.1371/journal.pcbi.1005416 (PMC5358897; doi:10.1371/journal.pcbi.1005416)
Supplement: S4 Text — (PDF) [file pcbi.1005416.s004.pdf]

S4 Text

Comparison between kernel distance and summary statistics distance.

We wanted to verify if the inference issues of the kernel-ABC were only related to the MCMC implementation, or if they could be also imputed to the distance itself. Thus we decided to use the kernel distance of [1, 2] in our simple rejection algorithm.

We wrote a python script, named `kernelDistance.py`, which contains functions extracted from `kamphir.py` and `phyloK2.py`, from the kamphir software package of [2]. That way, the `kernelDistance.py` script uses exactly the same distance computation algorithm as in the original kamphir software package of [2]. All the code is freely accessible from `http://dx.doi.org/10.6084/m9.figshare.4235354`. This script takes two inputs, the file containing the target tree (in Newick format) and the file containing the simulated trees for ABC (one line per tree in Newick format), and returns the vector of computed distances. This output file is then used as an input in the rejection algorithm.

Then, rejection here consists of selecting a proportion P_δ of the simulations for which the kernel-distance with the target tree is small. We used several tolerance values ($P_\delta = \{0.01; 0.05; 0.1; 0.2; 0.3; 0.4; 0.5\}$). We performed the same rejection with the Euclidean distance between vectors of 83 summary statistics.

References

[1] Poon AFY, Walker LW, Murray H, McCloskey RM, Harrigan PR, Liang RH. Mapping the shapes of phylogenetic trees from human and zoonotic RNA viruses. PLoS One. 2013;8(11):e78122. Available from: `http://dx.doi.org/10.1371/journal.pone.0078122`.

[2] Poon AFY. Phylodynamic Inference with Kernel ABC and Its Application to HIV Epidemiology. Mol Biol Evol. 2015 Sep;32(9):2483–2495. Available from: `http://dx.doi.org/10.1093/molbev/msv123`.
