# Supplementary material for: Inferring epidemiological parameters from phylogenies using regression-ABC: A comparative study
Source: PLoS Comput Biol. 2017 Mar 6;13(3):e1005416. doi: 10.1371/journal.pcbi.1005416 (PMC5358897; doi:10.1371/journal.pcbi.1005416)
Supplement: S5 Text — (PDF) [file pcbi.1005416.s005.pdf]

S5 Text

ABC-LASSO robustness analysis.

As the EBOV phylogeny from [1] is partially unresolved, we wanted to verify the robustness of our ABC-LASSO algorithm to the phylogenetic uncertainty about the tree topology.

To do so, we downloaded the sequence alignment from [1] and reproduced the phylogenetic inferences of [1] using RAxML v8.2 [2]. Indeed, as in [1], we ran fifty instances of RAxML inference from a random starting tree, using the GTRΓ nucleotide substitution model and the bootstrapping algorithm with 10 pseudoreplicates. Then instead of taking the best scoring tree, we kept the 10 best. RAxML likelihood scores associated with all trees were very close (the score of the phylogeny from Gire et al. is -35018.708 ; scores of the 10 best replicates lie between -35018.706 and -35018.701 ; and scores of the 50 replicates lie between -35018.828 and -35018.701). Our 10 best trees were time-scaled using LSD software, as the tree from Gire et al.. We computed all the summary statistics on the resulting time-scaled phylogenies and performed the same ABC-LASSO inferences as for the tree from Gire et al., using the same set of simulated trees for ABC.

We also computed the Robinson-Foulds (RF) bi-partition distance between the 10 newly inferred trees and the tree from Gire et al., to measure topological dissimilarities [3]. This was done using the program `treedist` from PHYLIP v3.696 [4]. We normalized the distance measurements by the maximal RF distance for a rooted tree: $\mathrm{RF}_{\max} = 2(n-2)$, where n is the number of taxa.

References

[1] Gire SK, Goba A, Andersen KG, Sealfon RSG, Park DJ, Kanneh L, et al. Genomic surveillance elucidates Ebola virus origin and transmission during the 2014 outbreak. Science. 2014 Sep;345(6202):1369–1372. Available from: http://dx.doi.org/10.1126/science.1259657.

[2] Stamatakis A. RAxML version 8: a tool for phylogenetic analysis and post-analysis of large phylogenies. Bioinformatics (Oxford, England). 2014 May;30:1312–1313.

[3] Robinson DF, Foulds LR. Comparison of phylogenetic trees. Mathematical biosciences. 1981;53(1-2):131–147.

[4] Felsenstein J. PHYLIP - Phylogeny Inference Package (Version 3.2). Cladistics. 1989;5:163–166.
