# Supplementary material for: Inferring epidemiological parameters from phylogenies using regression-ABC: A comparative study
Source: PLoS Comput Biol. 2017 Mar 6;13(3):e1005416. doi: 10.1371/journal.pcbi.1005416 (PMC5358897; doi:10.1371/journal.pcbi.1005416)
Supplement: S1 Table — (PDF) [file pcbi.1005416.s016.pdf]

S1 Table

Table of correlations between the summary statistics of the BL, LTT and TOPO sets and the epidemiological parameters of the SIR model, for trees of 100 leaves.

Summary statistics	Set	R_0	d_i	N	Sum
mean_s_time	LTT	−0.66	0.7	−0.02	1.4
slope_1	LTT	0.69	−0.68	0.01	1.4
slope_2	LTT	0.62	−0.73	0.03	1.4
t_max_L	LTT	−0.64	0.73	0	1.4
max_H	BL	−0.61	0.74	−0.02	1.4
mean_b_time[2]	LTT	−0.64	0.71	−0.02	1.4
i_BL_var_[2]	BL	−0.6	0.75	−0.01	1.4
e_BL_var	BL	−0.55	0.78	−0.02	1.4
i_BL_mean_[1]	BL	−0.62	0.72	−0.01	1.3
i_BL_var_[1]	BL	−0.61	0.72	−0.01	1.3
i_BL_mean_[2]	BL	−0.57	0.76	−0.01	1.3
i_BL_median_[2]	BL	−0.55	0.76	−0.02	1.3
i_BL_median_[1]	BL	−0.6	0.71	−0.01	1.3
a_BL_var	BL	−0.5	0.79	−0.02	1.3
i_BL_var_[3]	BL	−0.61	0.66	0.03	1.3
a_BL_mean	BL	−0.49	0.79	−0.01	1.3
mean_b_time[1]	LTT	−0.67	0.61	0.01	1.3
a_BL_median	BL	−0.48	0.79	−0.01	1.3
e_BL_mean	BL	−0.43	0.8	−0.02	1.2
i_BL_mean_[3]	BL	−0.5	0.73	0.01	1.2
i_BL_median_[3]	BL	−0.46	0.73	0.01	1.2
e_BL_median	BL	−0.39	0.79	−0.01	1.2
mean_b_time[3]	LTT	−0.45	0.65	0.02	1.1
min_H	BL	−0.37	0.72	0	1.1
max_L	LTT	0.63	−0.01	0.01	0.65
ie_BL_mean_[2]	BL	−0.56	0.01	0	0.57
ie_BL_mean_[1]	BL	−0.54	0.01	0.01	0.56
ie_BL_median_[2]	BL	−0.53	0.01	−0.01	0.55
ie_BL_median_[1]	BL	−0.5	0.01	0.01	0.52
slope_ratio	LTT	0.39	−0.01	−0.08	0.48
staircaseness_1	TOPO	0.37	0	0.01	0.38
ie_BL_var_[1]	BL	−0.34	0	0.03	0.37
ie_BL_var_[2]	BL	−0.32	0	0.03	0.35
sackin	TOPO	−0.33	0	0	0.33
IL_nodes	TOPO	−0.32	0	−0.01	0.33
ie_BL_var_[3]	BL	−0.26	0	0.05	0.31
staircaseness_2	TOPO	−0.28	0	−0.01	0.29
WD_ratio	TOPO	0.26	0.01	0	0.27
ie_BL_mean_[3]	BL	−0.25	0	−0.01	0.26
ie_BL_median_[3]	BL	−0.21	0	−0.01	0.22
Δw	TOPO	0.15	0	0.01	0.16
max_ladder	TOPO	−0.15	0	0	0.15
colless	TOPO	0.01	−0.01	0	0.02
