# Supplementary material for: Inferring epidemiological parameters from phylogenies using regression-ABC: A comparative study
Source: PLoS Comput Biol. 2017 Mar 6;13(3):e1005416. doi: 10.1371/journal.pcbi.1005416 (PMC5358897; doi:10.1371/journal.pcbi.1005416)
Supplement: S3 Table — (PDF) [file pcbi.1005416.s018.pdf]

S3 Table

Table of correlations between the summary statistics of the COORDS set and the epidemiological parameters of the SIR model, for trees of 100 leaves.

Coordinate	R_0	d_i	N	Sum
x_8	−0.65	0.72	0	1.4
x_9	−0.65	0.72	0	1.4
x_{11}	−0.64	0.73	0	1.4
x_{13}	−0.62	0.74	−0.01	1.4
x_{15}	−0.61	0.75	−0.01	1.4
x_{16}	−0.61	0.75	−0.01	1.4
x_{17}	−0.61	0.75	−0.01	1.4
x_{18}	−0.61	0.75	−0.01	1.4
x_{19}	−0.61	0.75	−0.01	1.4
x_4	−0.65	0.71	0	1.4
x_5	−0.65	0.71	0	1.4
x_6	−0.65	0.71	0	1.4
x_7	−0.65	0.71	0	1.4
x_{10}	−0.64	0.72	0	1.4
x_{12}	−0.62	0.74	0	1.4
x_{14}	−0.61	0.74	−0.01	1.4
x_{20}	−0.61	0.74	−0.01	1.4
x_3	−0.65	0.7	0	1.4
x_2	−0.64	0.7	0	1.3
x_1	−0.61	0.69	0	1.3
y_{11}	0.63	−0.01	0.01	0.65
y_{12}	0.63	−0.01	0.01	0.65
y_9	0.62	−0.01	0.01	0.64
y_{10}	0.62	−0.01	0.01	0.64
y_{13}	0.62	0	0.01	0.63
y_8	0.61	−0.01	0	0.62
y_{14}	0.61	0	0.01	0.62
y_6	0.59	−0.01	0.01	0.61
y_7	0.6	−0.01	0	0.61
y_5	0.58	−0.01	0.01	0.6
y_{15}	0.59	0	0.01	0.6
y_4	0.57	−0.01	0.01	0.59
y_{16}	0.57	0	0.01	0.58
y_3	0.54	0	0.01	0.55
y_{17}	0.53	0	0.01	0.54
y_2	0.5	0	0	0.5
y_{18}	0.46	0	0.01	0.47
y_1	0.41	0	0	0.41
y_{19}	0.37	0	0.01	0.38
y_{20}	0.24	0	0.01	0.25
