# Supplementary material for: Inferring epidemiological parameters from phylogenies using regression-ABC: A comparative study
Source: PLoS Comput Biol. 2017 Mar 6;13(3):e1005416. doi: 10.1371/journal.pcbi.1005416 (PMC5358897; doi:10.1371/journal.pcbi.1005416)
Supplement: S4 Table — (PDF) [file pcbi.1005416.s019.pdf]

S4 Table

Table of correlations between the summary statistics of the COORDS set and the epidemiological parameters of the SIR model, for trees of 1,000 leaves.

Coordinate	R_0	d_i	N	Sum
x_5	−0.67	0.71	0.02	1.4
x_9	−0.66	0.72	0.02	1.4
x_3	−0.67	0.7	0.02	1.4
x_4	−0.67	0.7	0.02	1.4
x_6	−0.66	0.71	0.02	1.4
x_7	−0.66	0.71	0.02	1.4
x_8	−0.66	0.71	0.02	1.4
x_{10}	−0.65	0.72	0.02	1.4
x_{11}	−0.64	0.73	0.02	1.4
x_2	−0.66	0.7	0.02	1.4
x_{12}	−0.63	0.73	0.02	1.4
x_{13}	−0.62	0.74	0.02	1.4
x_{15}	−0.61	0.75	0.02	1.4
x_1	−0.65	0.7	0.02	1.4
x_{14}	−0.61	0.74	0.02	1.4
x_{16}	−0.6	0.75	0.02	1.4
x_{17}	−0.59	0.75	0.01	1.3
x_{18}	−0.59	0.75	0.01	1.3
x_{19}	−0.58	0.75	0.01	1.3
x_{20}	−0.57	0.75	0	1.3
y_{14}	0.77	0	0.12	0.89
y_{15}	0.77	0	0.12	0.89
y_{16}	0.77	0	0.12	0.89
y_{13}	0.76	0	0.12	0.88
y_{11}	0.74	0	0.13	0.87
y_{12}	0.75	0	0.12	0.87
y_{10}	0.73	0	0.13	0.86
y_9	0.72	0	0.13	0.85
y_{17}	0.74	0	0.11	0.85
y_7	0.71	0	0.13	0.84
y_8	0.71	0	0.13	0.84
y_5	0.7	0	0.12	0.82
y_6	0.7	0	0.12	0.82
y_4	0.69	0	0.12	0.81
y_3	0.68	0	0.12	0.8
y_2	0.67	0	0.11	0.78
y_{18}	0.67	0	0.11	0.78
y_1	0.64	0	0.1	0.74
y_{19}	0.49	0	0.09	0.58
y_{20}	0.21	0	0.06	0.27
