# Supplementary material for: Inferring epidemiological parameters from phylogenies using regression-ABC: A comparative study
Source: PLoS Comput Biol. 2017 Mar 6;13(3):e1005416. doi: 10.1371/journal.pcbi.1005416 (PMC5358897; doi:10.1371/journal.pcbi.1005416)
Supplement: S7 Table — (PDF) [file pcbi.1005416.s022.pdf]

S7 Table

Table of correlations between the summary statistics of the COORDS set and the epidemiological parameters of the BD model, for trees of 100 leaves.

Coordinate	R_0	d_i	Sum
x_6	-0.65	0.71	1.4
x_7	-0.65	0.71	1.4
x_8	-0.65	0.71	1.4
x_9	-0.65	0.71	1.4
x_{10}	-0.64	0.72	1.4
x_{13}	-0.62	0.74	1.4
x_{18}	-0.62	0.74	1.4
x_{19}	-0.62	0.74	1.4
x_{20}	-0.62	0.74	1.4
x_3	-0.65	0.7	1.4
x_4	-0.65	0.7	1.4
x_5	-0.65	0.7	1.4
x_{11}	-0.63	0.72	1.4
x_{12}	-0.62	0.73	1.4
x_{14}	-0.61	0.74	1.4
x_{15}	-0.61	0.74	1.4
x_{16}	-0.61	0.74	1.4
x_{17}	-0.61	0.74	1.4
x_2	-0.64	0.7	1.3
x_1	-0.61	0.68	1.3
y_{11}	0.63	0.01	0.64
y_{12}	0.63	0.01	0.64
y_{13}	0.62	0.01	0.63
y_9	0.62	0	0.62
y_{10}	0.62	0	0.62
y_{14}	0.61	0.01	0.62
y_6	0.6	0.01	0.61
y_7	0.61	0	0.61
y_8	0.61	0	0.61
y_5	0.59	0.01	0.6
y_{15}	0.59	0.01	0.6
y_4	0.57	0.01	0.58
y_3	0.55	0.01	0.56
y_{16}	0.56	0	0.56
y_2	0.51	0.01	0.52
y_{17}	0.51	0	0.51
y_{18}	0.46	0	0.46
y_1	0.42	0.01	0.43
y_{19}	0.37	0	0.37
y_{20}	0.23	0	0.23
