# Supplementary material for: Inferring epidemiological parameters from phylogenies using regression-ABC: A comparative study
Source: PLoS Comput Biol. 2017 Mar 6;13(3):e1005416. doi: 10.1371/journal.pcbi.1005416 (PMC5358897; doi:10.1371/journal.pcbi.1005416)
Supplement: S8 Table — (PDF) [file pcbi.1005416.s023.pdf]

S8 Table

Table of correlations between the summary statistics of the COORDS set and the epidemiological parameters of the BD model, for trees of 1,000 leaves.

Coordinate	R_0	d_i	Sum
x_4	−0.67	0.71	1.4
x_5	−0.67	0.71	1.4
x_9	−0.66	0.72	1.4
x_2	−0.67	0.7	1.4
x_3	−0.67	0.7	1.4
x_6	−0.66	0.71	1.4
x_7	−0.66	0.71	1.4
x_8	−0.66	0.71	1.4
x_{10}	−0.65	0.72	1.4
x_{11}	−0.65	0.72	1.4
x_{12}	−0.64	0.73	1.4
x_{13}	−0.64	0.73	1.4
x_{14}	−0.64	0.73	1.4
x_{15}	−0.64	0.73	1.4
x_{16}	−0.64	0.73	1.4
x_{17}	−0.64	0.73	1.4
x_{18}	−0.64	0.73	1.4
x_{19}	−0.64	0.73	1.4
x_{20}	−0.64	0.73	1.4
x_1	−0.66	0.7	1.4
y_5	0.62	0	0.62
y_6	0.62	0	0.62
y_7	0.62	0	0.62
y_8	0.62	0	0.62
y_9	0.62	0	0.62
y_{10}	0.62	0	0.62
y_{11}	0.62	0	0.62
y_{12}	0.62	0	0.62
y_{13}	0.62	0	0.62
y_3	0.61	0	0.61
y_4	0.61	0	0.61
y_{14}	0.61	0	0.61
y_2	0.6	0	0.6
y_{15}	0.6	0	0.6
y_{16}	0.59	0	0.59
y_1	0.58	0	0.58
y_{17}	0.55	0	0.55
y_{18}	0.49	0	0.49
y_{19}	0.35	0	0.35
y_{20}	0.12	0	0.12
