# Supplementary material for: Inferring epidemiological parameters from phylogenies using regression-ABC: A comparative study
Source: PLoS Comput Biol. 2017 Mar 6;13(3):e1005416. doi: 10.1371/journal.pcbi.1005416 (PMC5358897; doi:10.1371/journal.pcbi.1005416)
Supplement: S9 Table — (PDF) [file pcbi.1005416.s024.pdf]

S9 Table

Table of correlations between the summary statistics of the BL, TOPO and LTT sets and the epidemiological parameters of the SI-DR model, for ultrametric trees of 300 leaves.

Summary statistics	Set	c_1	β	γ	N	Sum
ie_BL_median_[2]	BL	0.32	0.2	0.69	−0.23	1.4
ie_BL_mean_[2]	BL	0.32	0.21	0.66	−0.22	1.4
ie_BL_mean_[3]	BL	0.27	0.19	0.65	−0.24	1.4
ie_BL_median_[3]	BL	0.26	0.17	0.67	−0.24	1.3
i_BL_var_[2]	BL	0.45	0.35	0.41	−0.13	1.3
i_BL_var_[3]	BL	0.37	0.34	0.36	−0.12	1.2
e_BL_mean	BL	0.13	0.25	−0.53	0.23	1.1
a_BL_mean	BL	0.21	0.3	−0.43	0.19	1.1
e_BL_median	BL	0.09	0.23	−0.57	0.23	1.1
ie_BL_mean_[1]	BL	−0.11	−0.34	0.45	−0.21	1.1
a_BL_median	BL	0.14	0.22	−0.52	0.22	1.1
ie_BL_median_[1]	BL	−0.18	−0.4	0.35	−0.17	1.1
a_BL_var	BL	0.27	0.36	−0.31	0.15	1.1
i_BL_mean_[3]	BL	0.21	0.32	−0.38	0.17	1.1
mean_b_time[3]	LTT	0.04	0.17	−0.62	0.25	1.1
i_BL_median_[3]	BL	0.17	0.3	−0.42	0.17	1.1
t_max_L	LTT	0.55	0.17	0.22	−0.09	1
slope_1	LTT	−0.55	−0.17	−0.22	0.09	1
mean_b_time[1]	LTT	−0.11	−0.33	0.41	−0.18	1
e_BL_var	BL	0.34	0.35	0.22	−0.08	0.99
i_BL_var_[1]	BL	0.29	−0.07	0.42	−0.19	0.97
i_BL_median_[2]	BL	0.41	0.44	0.05	0.03	0.93
slope_ratio	LTT	−0.06	0.07	−0.55	0.23	0.91
i_BL_mean_[1]	BL	−0.06	−0.46	0.24	−0.15	0.91
i_BL_mean_[2]	BL	0.41	0.43	0.03	0.03	0.9
slope_2	LTT	0.03	−0.07	0.54	−0.23	0.87
i_BL_median_[1]	BL	−0.25	−0.52	−0.02	−0.04	0.83
min_H	BL	0.56	0.21	0.03	0	0.8
max_H	BL	0.56	0.21	0.03	0	0.8
mean_b_time[2]	LTT	0.29	0.32	−0.1	0.08	0.79
mean_s_time	LTT	0.24	0.23	−0.11	0.04	0.62
ie_BL_var_[3]	BL	0.1	−0.02	0.37	−0.12	0.61
ie_BL_var_[1]	BL	−0.06	−0.29	0.11	−0.05	0.51
ie_BL_var_[2]	BL	0.13	−0.03	0.21	−0.04	0.41
sackin	TOPO	−0.11	0.1	−0.05	−0.01	0.27
colless	TOPO	−0.08	0.07	−0.03	−0.01	0.19
WD_ratio	TOPO	0.07	−0.07	0.04	0.01	0.19
Δw	TOPO	0.04	−0.04	0.02	0	0.1
staircaseness_1	TOPO	0.02	−0.02	0.01	0.02	0.07
staircaseness_2	TOPO	0	0	0.01	−0.02	0.03
max_ladder	TOPO	0.01	0	0.01	0	0.02
IL_nodes	TOPO	0	0	0	−0.01	0.01
