# Supplementary material for: Inferring epidemiological parameters from phylogenies using regression-ABC: A comparative study
Source: PLoS Comput Biol. 2017 Mar 6;13(3):e1005416. doi: 10.1371/journal.pcbi.1005416 (PMC5358897; doi:10.1371/journal.pcbi.1005416)
Supplement: S10 Table — (PDF) [file pcbi.1005416.s025.pdf]

S10 Table

Table of correlations between the summary statistics of the BL, TOPO and LTT sets and the epidemiological parameters of the SI-DR model, for non-ultrametric trees of 300 leaves.

Summary statistics	Set	c_1	β	γ	N	Sum
ie_BL_mean_[2]	BL	0.39	0.32	0.47	−0.16	1.3
ie_BL_median_[2]	BL	0.36	0.32	0.48	−0.15	1.3
i_BL_var_[2]	BL	0.44	0.35	0.32	−0.12	1.2
i_BL_var_[3]	BL	0.29	0.17	0.49	−0.21	1.2
ie_BL_var_[2]	BL	0.35	0.18	0.43	−0.17	1.1
e_BL_mean	BL	0.26	0.34	−0.32	0.15	1.1
e_BL_median	BL	0.25	0.34	−0.33	0.15	1.1
ie_BL_mean_[1]	BL	−0.17	−0.39	0.32	−0.17	1
a_BL_mean	BL	0.32	0.37	−0.23	0.11	1
a_BL_var	BL	0.31	0.38	−0.22	0.12	1
i_BL_var_[1]	BL	0.36	−0.04	0.42	−0.21	1
slope_1	LTT	−0.06	0.29	−0.45	0.21	1
ie_BL_var_[3]	BL	0.18	0.04	0.56	−0.23	1
i_BL_mean_[1]	BL	0.09	−0.41	0.31	−0.19	1
ie_BL_median_[1]	BL	−0.28	−0.46	0.15	−0.1	0.99
a_BL_median	BL	0.34	0.36	−0.19	0.09	0.98
slope_2	LTT	0	0.17	−0.57	0.23	0.97
mean_b_time[1]	LTT	−0.1	−0.37	0.33	−0.17	0.97
mean_b_time[2]	LTT	0.24	0.34	−0.26	0.13	0.97
e_BL_var	BL	0.39	0.36	0.15	−0.06	0.96
i_BL_mean_[2]	BL	0.37	0.41	−0.06	0.05	0.89
i_BL_median_[2]	BL	0.36	0.41	−0.05	0.05	0.87
min_H	BL	0.55	0.21	0.04	−0.03	0.83
max_H	BL	0.55	0.21	0.04	−0.03	0.83
t_max_L	LTT	0.32	−0.21	0.16	−0.12	0.81
i_BL_median_[1]	BL	−0.18	−0.52	−0.03	−0.06	0.79
ie_BL_var_[1]	BL	0.03	−0.25	0.33	−0.17	0.78
i_BL_mean_[3]	BL	0.22	0.29	−0.17	0.08	0.76
slope_ratio	LTT	−0.11	0.29	−0.21	0.12	0.73
i_BL_median_[3]	BL	0.19	0.28	−0.18	0.08	0.73
IL_nodes	TOPO	−0.11	−0.16	0.16	−0.06	0.49
staircaseness_1	TOPO	0.14	0.14	−0.15	0.05	0.48
staircaseness_2	TOPO	−0.11	−0.15	0.14	−0.06	0.46
mean_b_time[3]	LTT	0.03	0.13	−0.15	0.03	0.34
max_ladder	TOPO	−0.06	−0.09	0.11	−0.05	0.31
ie_BL_median_[3]	BL	−0.15	0.01	0.07	−0.02	0.25
sackin	TOPO	−0.22	−0.01	−0.01	0	0.24
WD_ratio	TOPO	0.18	0.03	−0.02	0	0.23
Δw	TOPO	0.13	0.05	−0.02	0.02	0.22
ie_BL_mean_[3]	BL	−0.05	0.08	0.07	−0.01	0.21
colless	TOPO	−0.1	0.04	−0.04	0.01	0.19
mean_s_time	LTT	0.01	0.01	−0.02	0	0.04
