# Supplementary material for: Inferring epidemiological parameters from phylogenies using regression-ABC: A comparative study
Source: PLoS Comput Biol. 2017 Mar 6;13(3):e1005416. doi: 10.1371/journal.pcbi.1005416 (PMC5358897; doi:10.1371/journal.pcbi.1005416)
Supplement: S11 Table — (PDF) [file pcbi.1005416.s026.pdf]

S11 Table

Table of correlations between the summary statistics of the COORDS set and the epidemiological parameters of the SI-DR model, for ultrametric trees of 300 leaves.

Coordinate	c_1	β	γ	N	Sum
x_{14}	0.42	0.17	0.62	−0.24	1.4
x_{13}	0.28	0	0.76	−0.3	1.3
x_{12}	0.18	−0.1	0.73	−0.29	1.3
x_{11}	0.1	−0.18	0.67	−0.27	1.2
x_{10}	0.03	−0.24	0.59	−0.24	1.1
x_9	−0.03	−0.29	0.51	−0.21	1
x_{15}	0.5	0.32	0.17	−0.05	1
x_8	−0.09	−0.33	0.42	−0.18	1
x_7	−0.15	−0.36	0.33	−0.15	0.99
x_1	−0.41	−0.44	−0.1	0	0.95
x_6	−0.19	−0.39	0.25	−0.12	0.95
y_1	0.25	0.37	−0.22	0.11	0.95
y_2	0.25	0.37	−0.22	0.11	0.95
y_3	0.25	0.37	−0.22	0.11	0.95
y_4	0.25	0.37	−0.22	0.11	0.95
y_5	0.25	0.37	−0.22	0.11	0.95
y_6	0.25	0.37	−0.22	0.11	0.95
y_7	0.25	0.37	−0.22	0.11	0.95
y_8	0.25	0.37	−0.22	0.11	0.95
y_9	0.25	0.37	−0.22	0.11	0.95
y_{10}	0.25	0.37	−0.22	0.11	0.95
y_{11}	0.25	0.37	−0.22	0.11	0.95
y_{12}	0.25	0.37	−0.22	0.11	0.95
y_{13}	0.25	0.37	−0.22	0.11	0.95
y_{14}	0.25	0.37	−0.22	0.11	0.95
x_5	−0.24	−0.41	0.17	−0.1	0.92
x_2	−0.37	−0.46	−0.05	−0.03	0.91
x_{16}	0.54	0.29	0.06	0	0.89
x_4	−0.29	−0.43	0.09	−0.07	0.88
x_{17}	0.55	0.26	0.06	0	0.87
y_{19}	−0.21	−0.32	0.21	−0.11	0.85
x_3	−0.33	−0.45	0.01	−0.05	0.84
y_{20}	−0.18	−0.29	0.25	−0.12	0.84
x_{18}	0.56	0.22	0.04	0	0.82
y_{16}	0.27	0.28	0.21	−0.06	0.82
x_{19}	0.56	0.21	0.03	0	0.8
x_{20}	0.56	0.21	0.03	0	0.8
y_{15}	0.24	0.36	−0.13	0.07	0.8
y_{17}	0.14	0.22	0.01	0.01	0.38
y_{18}	−0.04	−0.03	0.06	−0.01	0.14
