# Supplementary material for: Inferring epidemiological parameters from phylogenies using regression-ABC: A comparative study
Source: PLoS Comput Biol. 2017 Mar 6;13(3):e1005416. doi: 10.1371/journal.pcbi.1005416 (PMC5358897; doi:10.1371/journal.pcbi.1005416)
Supplement: S12 Table — (PDF) [file pcbi.1005416.s027.pdf]

S12 Table

Table of correlations between the summary statistics of the COORDS set and the epidemiological parameters of the SI-DR model, for non-ultrametric trees of 300 leaves.

Coordinate	c_1	β	γ	N	Sum
x_{10}	0.26	−0.19	0.56	−0.27	1.3
x_{11}	0.48	−0.07	0.44	−0.22	1.2
x_{14}	0.55	0.05	0.37	−0.18	1.2
x_{13}	0.53	0.01	0.39	−0.19	1.1
x_{15}	0.57	0.11	0.3	−0.14	1.1
x_9	0.09	−0.28	0.5	−0.24	1.1
x_{12}	0.51	−0.02	0.39	−0.19	1.1
y_{12}	0.09	0.23	−0.53	0.23	1.1
y_{13}	0.07	0.22	−0.55	0.24	1.1
y_{10}	0.12	0.26	−0.48	0.21	1.1
y_{11}	0.11	0.24	−0.5	0.22	1.1
x_{16}	0.57	0.16	0.22	−0.11	1.1
y_{14}	0.05	0.2	−0.57	0.24	1.1
y_{15}	0.04	0.18	−0.58	0.24	1
x_1	−0.41	−0.46	−0.12	0.01	1
x_{17}	0.57	0.18	0.17	−0.08	1
y_{16}	0.02	0.16	−0.58	0.24	1
x_8	−0.02	−0.34	0.4	−0.2	0.96
x_{18}	0.57	0.19	0.13	−0.07	0.96
y_{17}	0.01	0.15	−0.57	0.23	0.96
y_9	0.14	0.27	−0.38	0.16	0.95
x_2	−0.37	−0.48	−0.09	−0.01	0.95
x_7	−0.1	−0.39	0.3	−0.16	0.95
x_6	−0.17	−0.42	0.2	−0.12	0.91
x_{19}	0.56	0.21	0.07	−0.05	0.89
y_{18}	0.01	0.13	−0.54	0.21	0.89
x_3	−0.33	−0.47	−0.04	−0.03	0.87
x_5	−0.23	−0.44	0.1	−0.08	0.85
x_{20}	0.55	0.21	0.04	−0.03	0.83
x_4	−0.28	−0.46	0.03	−0.05	0.82
y_8	0.16	0.27	−0.27	0.11	0.81
y_{19}	0	0.12	−0.47	0.18	0.77
y_7	0.19	0.27	−0.18	0.08	0.72
y_6	0.22	0.26	−0.11	0.04	0.63
y_5	0.24	0.25	−0.06	0.02	0.57
y_{20}	0	0.08	−0.32	0.13	0.53
y_4	0.24	0.24	−0.02	0.01	0.51
y_3	0.24	0.23	0	−0.01	0.48
y_2	0.23	0.16	−0.03	−0.02	0.44
y_1	0.13	−0.02	−0.12	0	0.27
