# Supplementary material for: Inferring epidemiological parameters from phylogenies using regression-ABC: A comparative study
Source: PLoS Comput Biol. 2017 Mar 6;13(3):e1005416. doi: 10.1371/journal.pcbi.1005416 (PMC5358897; doi:10.1371/journal.pcbi.1005416)
Supplement: S13 Table — (PDF) [file pcbi.1005416.s028.pdf]

S13 Table

Table of correlations between the summary statistics of the BL, LTT and TOPO sets and the epidemiological parameters of the BDEI model, for trees of 72 leaves simulated assuming p ≈ 0.4.

Summary statistics	Set	R_0	d_E	d_I	Sum
max_H	BL	−0.64	0.22	0.49	1.4
e_BL_var	BL	−0.44	0.43	0.47	1.3
i_BL_mean_[2]	BL	−0.55	0.37	0.41	1.3
i_BL_var_[2]	BL	−0.56	0.41	0.36	1.3
t_max_L	LTT	−0.62	0.18	0.47	1.3
slope_1	LTT	0.7	−0.13	−0.44	1.3
mean_b_time[2]	LTT	−0.69	0.17	0.4	1.3
i_BL_var_[3]	BL	−0.52	0.37	0.37	1.3
i_BL_mean_[1]	BL	−0.6	0.18	0.43	1.2
i_BL_var_[1]	BL	−0.53	0.29	0.39	1.2
i_BL_median_[2]	BL	−0.48	0.3	0.42	1.2
slope_2	LTT	0.54	−0.2	−0.41	1.2
a_BL_var	BL	−0.29	0.43	0.38	1.1
mean_s_time	LTT	−0.49	0.17	0.44	1.1
mean_b_time[1]	LTT	−0.59	−0.05	0.43	1.1
i_BL_mean_[3]	BL	−0.25	0.48	0.34	1.1
i_BL_median_[1]	BL	−0.58	0.04	0.42	1
a_BL_mean	BL	−0.22	0.41	0.39	1
ie_BL_median_[1]	BL	−0.62	−0.17	0.23	1
i_BL_median_[3]	BL	−0.13	0.52	0.29	0.94
a_BL_median	BL	−0.14	0.42	0.37	0.93
ie_BL_mean_[1]	BL	−0.62	−0.1	0.21	0.93
ie_BL_median_[2]	BL	−0.65	−0.08	0.18	0.91
mean_b_time[3]	LTT	−0.21	0.36	0.33	0.9
max_L	LTT	0.56	−0.02	−0.24	0.82
e_BL_median	BL	0.12	0.38	0.29	0.79
ie_BL_mean_[2]	BL	−0.66	−0.01	0.12	0.79
e_BL_mean	BL	−0.02	0.4	0.33	0.75
min_H	BL	−0.34	0.12	0.22	0.68
ie_BL_var_[2]	BL	−0.35	0.13	−0.08	0.56
ie_BL_mean_[3]	BL	−0.41	0.15	0	0.56
ie_BL_median_[3]	BL	−0.37	0.18	0.01	0.56
staircaseness_1	Topo	0.13	−0.29	0.08	0.5
ie_BL_var_[1]	BL	−0.34	−0.02	0.11	0.47
staircaseness_2	Topo	−0.15	0.24	−0.07	0.46
IL_nodes	Topo	−0.2	0.21	−0.03	0.44
ie_BL_var_[3]	BL	−0.29	0.04	−0.08	0.41
WD_ratio	Topo	0.08	−0.22	0.08	0.38
max_ladder	Topo	−0.09	0.2	−0.08	0.37
slope_ratio	LTT	−0.04	0.2	0.1	0.34
sackin	Topo	−0.15	0.14	−0.01	0.3
Δw	Topo	0.04	−0.17	0.08	0.29
colless	Topo	0.07	0.09	−0.05	0.21
