# Supplementary material for: Inferring epidemiological parameters from phylogenies using regression-ABC: A comparative study
Source: PLoS Comput Biol. 2017 Mar 6;13(3):e1005416. doi: 10.1371/journal.pcbi.1005416 (PMC5358897; doi:10.1371/journal.pcbi.1005416)
Supplement: S14 Table — (PDF) [file pcbi.1005416.s029.pdf]

S14 Table

Table of correlations between the summary statistics of the COORDS set and the epidemiological parameters of the BDEI model, for trees of 72 leaves simulated assuming p $\approx$ 0.4.

Coordinate	R_0	d_E	d_I	Sum
x_{20}	−0.63	0.25	0.48	1.4
x_{18}	−0.62	0.25	0.48	1.4
x_{19}	−0.62	0.25	0.48	1.4
x_{16}	−0.61	0.25	0.48	1.3
x_{17}	−0.61	0.25	0.48	1.3
x_8	−0.68	0.19	0.46	1.3
x_9	−0.67	0.2	0.46	1.3
x_{10}	−0.65	0.21	0.46	1.3
x_{15}	−0.6	0.25	0.47	1.3
x_7	−0.68	0.17	0.46	1.3
x_6	−0.68	0.16	0.46	1.3
x_{14}	−0.6	0.24	0.46	1.3
x_5	−0.68	0.15	0.46	1.3
x_{13}	−0.59	0.24	0.46	1.3
x_4	−0.68	0.14	0.46	1.3
x_{12}	−0.59	0.23	0.45	1.3
x_{11}	−0.59	0.23	0.44	1.3
x_3	−0.67	0.12	0.46	1.2
x_2	−0.66	0.08	0.46	1.2
x_1	−0.6	0.01	0.46	1.1
y_{11}	0.57	−0.02	−0.23	0.82
y_{12}	0.59	−0.01	−0.21	0.81
y_{13}	0.6	−0.01	−0.2	0.81
y_{10}	0.55	−0.01	−0.24	0.8
y_{14}	0.61	−0.01	−0.18	0.8
y_9	0.53	−0.01	−0.25	0.79
y_{15}	0.61	−0.01	−0.17	0.79
y_8	0.51	−0.01	−0.26	0.78
y_7	0.48	0	−0.27	0.75
y_{16}	0.6	0	−0.15	0.75
y_6	0.46	0	−0.27	0.73
y_5	0.43	0	−0.28	0.71
y_4	0.4	0.01	−0.28	0.69
y_{17}	0.57	0	−0.12	0.69
y_3	0.37	0.02	−0.27	0.66
y_2	0.32	0.04	−0.26	0.62
y_{18}	0.5	0.01	−0.09	0.6
y_1	0.24	0.07	−0.19	0.5
y_{19}	0.37	0.03	−0.06	0.46
y_{20}	0.14	0.01	−0.02	0.17
